# Liquid-Crystal Ordering and Microphase Separation in the Lamellar Phase of Rod-Coil-Rod Triblock Copolymers. Molecular Theory and Computer Simulations

**DOI:** 10.3390/polym13193392

**Published:** 2021-10-02

**Authors:** Mikhail A. Osipov, Maxim V. Gorkunov, Alexander A. Antonov, Anatoly V. Berezkin, Yaroslav V. Kudryavtsev

**Affiliations:** 1 Department of Mathematics and Statistics, University of Strathclyde, Glasgow G1 1XH, UK; 2 Topchiev Institute of Petrochemical Synthesis, Russian Academy of Sciences, 119991 Moscow, Russia; gorkunov@crys.ras.ru (M.V.G.); antonov.wasd@yandex.ru (A.A.A.); berezkin.anatoly@rambler.ru (A.V.B.); yar@ips.ac.ru (Y.V.K.); 3 Shubnikov Institute of Crystallography, Federal Scientific Research Centre “Crystallography and Photonics”, Russian Academy of Sciences, 119333 Moscow, Russia; 4 Frumkin Institute of Physical Chemistry and Electrochemistry, Russian Academy of Sciences, 119071 Moscow, Russia

**Keywords:** phase transitions, block copolymers, liquid crystals, microphase separation

## Abstract

A molecular model of the orientationally ordered lamellar phase exhibited by asymmetric rod-coil-rod triblock copolymers has been developed using the density-functional approach and generalizing the molecular-statistical theory of rod-coil diblock copolymers. An approximate expression for the free energy of the lamellar phase has been obtained in terms of the direct correlation functions of the system, the Flory-Huggins parameter and the Maier-Saupe orientational interaction potential between rods. A detailed derivation of several rod-rod and rod-coil density-density correlation functions required to evaluate the free energy is presented. The orientational and translational order parameters of rod and coil segments depending on the temperature and triblock asymmetry have been calculated numerically by direct minimization of the free energy. Different structure and ordering of the lamellar phase at high and low values of the triblock asymmetry is revealed and analyzed in detail. Asymmetric rod-coil-rod triblock copolymers have been simulated using the method of dissipative particle dynamics in the broad range of the Flory-Huggins parameter and for several values of the triblock asymmetry. It has been found that the lamellar phase appears to be the most stable one at strong segregation. The density distribution of the coil segments and the segments of the two different rods have been determined for different values of the segregation strength. The simulations confirm the existence of a weakly ordered lamellar phase predicted by the density-functional theory, in which the short rods separate from the long ones and are characterized by weak positional ordering.

## 1. Introduction

Rod-coil block copolymers are very interesting soft matter systems which combine the properties of coil-coil block copolymers and liquid crystals. They exhibit a large variety of anisotropic phases which are characterized by different types of translational and orientational ordering and are considered to be promising materials for applications in polymer photovoltaics, LEDs, thin-film transistors, and sensors [1,2,3,4] not to mention a plethora of tunable micellar structures in solutions [5]. Such copolymers are composed of flexible and rigid fragments of various chemical structure. Their rigid anisotropic fragments can be orientationally ordered both due to the anisotropic interaction between them (π−π conjugation, *H*-bonding, etc.) and due to the intrinsic macroscopic anisotropy of the separated domains. The most stable phase exhibited by rod-coil copolymers is the orthogonal lamellar phase which has exactly the same symmetry as the smectic A liquid crystal phase [6].

From the materials science point of view, triblock copolymers attract significant attention because of their architecture, which enables one to tune the macroscopic properties by changing the position, structure, and length of the third block [7,8]. In contrast to diblock copolymers, in the lamellar phase the triblock macromolecules can be in the looped and bridged configurations. In the looped case, the terminal blocks belong to the same layer, while in the bridged one, the tails reside in two different layers separated by a domain occupied by middle blocks. The presence of such bridges strongly affects the mechanical properties of block copolymer materials including, for example, thermoplastic elastomers [9]. It should be also noted that the overall structure of coil-rod-coil triblock copolymers is most reminiscent of conventional liquid crystals which usually possess a rod-like rigid core and two flexible tails. At the same time, the triblock macromolecules are significantly larger than typical low-molar-mass mesogenic molecules and the flexible blocks are substantially longer.

So far the statistical theory of triblock copolymers has been developed using two different approaches. The first approach is based on the Landau—de Gennes expansion of the free energy in terms of the translational order parameters [10,11,12]. The coefficients of such an expansion have been evaluated in terms of the monomer-monomer correlation functions of the ideal (Gaussian) polymer chains derived following the approach proposed by Leibler [13]. This theory enables one to describe various non-conventional morphologies, which have been overlooked in other approaches. One notes, however, that this approach is valid in the vicinity of the transition into the isotropic disordered phase. The equilibrium monomer density also contains only one Fourier harmonic and hence it can be used in the case of weak segregation only [14]. Another approach employs the self-consistent field theory (SCFT) which has been successfully used for the description of coil-coil and rod-coil diblock copolymers [15,16,17,18,19,20,21,22]. In this theory, the free energy of a single chain in a self-consistent mean-field is calculated by numerically evaluating the path integral along the chain or by solving the generalized diffusion equations for several joint worm-like chains. So far, SCFT based on path integral calculation has been applied only to the coil-coil-coil and coil-rod-coil triblock copolymers [23].

Another version of SCFT based on the model or worm-like chains has been also applied to rod-coil triblock copolymers [20,21,24,25,26,27,28,29,30,31]. One notes that even with recently developed effective numerical algorithms [22,32], SCFT remains computationally challenging particularly in the case of long triblock macromolecules with rigid fragments characterized by many orientational and translational degrees of freedom. As a result, the existing theory of rod-coil triblock copolymers employs a crude lattice model and does not take into consideration the orientational interaction between rigid fragments. The theory also does not describe the orientational ordering in rod-coil triblock copolymers [33]. Solution-based systems containing copolymers with rod-like blocks demonstrate even more complex behavior which stimulates using simplified scaling approaches [34]. SCFT has also been used to describe chain folding morphology of semicrystalline polymers based on a rod–coil multiblock model [35]. Molecular dynamics simulations have also been performed by Wilson et al. [36,37] in an isothermal-isobaric ensemble where the rigid block was represented by a spherocylinder and the coil was modeled by a sequence of tangential spheres. It has been shown that multiblock rod-coil copolymers can self-assemble into more complex structures, especially in solutions, where the simulations predicted formation of nanowires [37].

Recently, the general density functional approach, which is successfully used in the molecular theory of liquid crystals [38,39,40,41,42], has been applied by the authors to develop a molecular statistical theory of rod-coil diblock copolymers [43,44,45]. In this approach, the free energy is expressed as a functional of the equilibrium densities of rod and coil monomer units. Integral equations for these densities are obtained by minimization of the free energy functional. One notes that the density functional theory is not based on the expansion in terms of the order parameters. The monomer densities are nonlinear functions of the order parameters and hence contain an infinite number of Fourier harmonics. Thus the theory is expected to be approximately valid also in the case of relatively strong segregation far from the transition into the disordered phase. At the same time, the theory also employs the density-density correlation functions for Gaussian chains which are also used in the Landau—de Gennes theory of rod-coil block copolymers [46]. Such a density functional theory is not as precise as the full SCFT, but it is also much less computationally challenging and can be used to calculate detailed orientational and translational order parameter profiles in an efficient way [43,44].

Coarse-grained computer simulations of triblock copolymers are most popular for mapping out the morphological phase diagrams in solutions and for identifying potentially interesting aggregate structures [47,48]. Self-assembly of rod-coil-rod triblocks in rod- or coil-selective solvents can be predicted with Langevin dynamics [49] or dissipative particle dynamics (DPD) [50]. DPD simulations can be specific to the chemical nature of copolymers, for example, the flow behavior of pluronics [51] and the effect of doping of polypeptide rod-coil-rod copolymers with Au nanoparticles [52] can be analyzed. Microphase separation of triblock copolymers in the bulk can be effectively addressed provided that they contain no rigid blocks [53], while the studies of roil-coil-rod systems are still scarce since rearrangements of stacked rods take a lot of computation time in the absence of solvent. After the first Monte Carlo simulation of rod-coil-rod and coil-rod-coil aggregation in thin films [54], only two papers have been published up to date: A DPD study of rod-coil-rod copolymer self-assembly within a planar slit [55], where parallel half-cylinders and arrowhead-shaped morphology appeared for the copolymers with long symmetric rods, and a Brownian dynamics study [56], in which liquid crystalline ordering which leads to the hierarchical lamellar-in-lamella structures peculiar to such copolymers was addressed. In the present study, we simulate asymmetric rod-coil-rod triblock copolymers using the DPD technique previously applied by us for the investigation of the tilted phase in rod-coil diblock copolymers [45,57].

The paper is arranged as follows. In Section 2 we derive a molecular-statistical theory of rod-coil-rod triblock copolymers using the density functional approach and present an approximate expression for the free energy of the lamellar phase suitable for a numerical minimization procedure. The detailed derivation of the free energy is given in Appendix A. In Section 3, the Ornstein-Zernike equations for triblock copolymers are considered in order to establish a relation between the direct correlation functions which enter the expression for the free energy and the total pair correlation functions which can be calculated based on the statistics of Gaussian chains and rigid rods. The general derivation of the the Ornstein-Zernike equations is presented in Appendix B and the approximate solution of these equations is considered in Appendix C. Finally, we present in Section 3 explicit expressions for all density-density correlation functions of the system of noninteracting rod-coil-rod triblock molecules. A detailed derivation of these correlation functions is given in Appendix D. In Section 4.1 the results of numerical free energy minimization are presented including the phase diagrams, colormaps and profiles of orientational and translational order parameters in the lamellar phase. The results of DPD simulations of asymmetric rod-coil-rod triblock copolymers are presented in Section 4.2, and, as discussed in Section 4.3, they confirm the molecular-statistical theory predictions. Main conclusions are drawn in Section 5.

## 2. Molecular-Statistical Theory of Triblock Copolymers

### 2.1. General Density Functional Theory

The classical density functional approach has been developed in the statistical theory of inhomogeneous and anisotropic fluids and applied to the theory of liquid crystals [38,39,41,42,58,59]. Recently, the density functional approach has been used by the authors to develop a molecular theory of rod-coil diblock copolymers [43,44,45,60]. In this approach, the fluid is described by the Helmholtz free energy functional which depends on the particle density in phase space. In the case of triblock rod-coil-rod copolymers, the free energy functional depends on the equilibrium densities of rod and coil monomers and is related to the Gibbs free energy (which depends on the external fields) by the generalized Legendre transformation equation:

(1) $$\begin{array}{c} F\left[\rho_{r1}\left(x\right),\rho_{r2}\left(x\right),\rho_{c}\left(r\right)\right]=F\left[U_{r1}\left(x\right),U_{r2}\left(x\right),U_{c}\left(r\right)\right]\\ \;-\int \rho_{r1}\left(x\right)U_{r1}\left(x\right)dx-\int \rho_{r2}\left(x\right)U_{r2}\left(x\right)dx-\int \rho_{c}\left(r\right)U_{c}\left(r\right)dr, \end{array}$$

where $F[U_{r1}\left(x\right),U_{r2}\left(x\right),U_{c}\left(r\right)]$ is the Gibbs free energy which depends on the external fields $U_{\nu }$, ν=(r1,r2,c), acting on the rod and coil monomers. Here $\rho_{r1}\left(x\right),\rho_{r2}\left(x\right),\rho_{c}\left(r\right)$ are the phase space number densities of the rod 1, rod 2 and coil monomers, respectively, and the variable x=(r,a) denotes both the position r of a rod monomer and its orientation specified by the unit vector a pointing along the rod axis.

The equilibrium monomer densities in the phase space are formally defined as:

(2) $$\rho_{\nu }\left(x\right)=\langle \sum_{i}\delta \left(x-x_{i}\right)\rangle ,$$

where 〈⋯〉 denotes the ensemble average and the sum is over all monomers of the type ν. One notes that in contrast to rod monomers, the number density of coil monomers depends only on the position r.

The free energy functional $F[\rho_{r1}\left(x\right),\rho_{r2}\left(x\right),\rho_{c}\left(r\right)]$ of a block copolymer can generally be written as a sum of two terms:

(3) F=W+H,

where *W* is the free energy of the system without intermolecular interactions, and *H* depends on the intermolecular interactions and correlations. Without the external field, the functional *W* can be expressed as

(4) $$\begin{array}{c} \beta W\left[\rho_{r1}\left(x\right),\rho_{r2}\left(x\right),\rho_{c}\left(r\right)\right]=\int \rho_{r1}\left(x\right)\ln\left[\rho_{r1}\left(x\right)\Lambda \right]\;dx\\ \;+\int \rho_{r2}\left(x\right)\ln\left[\rho_{r2}\left(x\right)\Lambda \right]\;dx+\int \rho_{c}\left(r\right)\ln\left[\rho_{c}\left(r\right)\Lambda \right]\;dr, \end{array}$$

where $\beta ={\left(k_{B}T\right)}^{-1}$.

One notes that *W* is the orientational and translational entropy of the system which depends on the one-particle distribution functions $f_{\nu }\left(x\right)=\rho_{\nu }\left(x\right)/N_{\nu }$ that specify the probability to find a monomer of the type ν at the position r with the orientation a.

The second functional derivatives of the reduced free energy $H[\rho_{\nu }\left(x\right)]$ with respect to the equilibrium densities are related to the direct correlation functions of the system:

(5) $$\beta \frac{\delta^{2}H}{\delta \rho_{\nu }\left(x_{1}\right)\delta \rho_{\mu }\left(x_{2}\right)}=-C_{\nu \mu }\left(x_{1},x_{2}\right),$$

where $C_{\nu \mu }\left(x_{1},x_{2}\right)$ are the direct pair correlation functions between the monomers ν and μ, (ν,μ)=(r1,r2,c).

Now the free energy of the anisotropic phase can be obtained by performing a functional Taylor expansion of the free energy around its value in the isotropic phase of the copolymer. Similarly to the statistical theory of diblock copolymers [43,44,45], the free energy functional of the rod-coil-rod triblock copolymer can approximately be expressed as:

(6) $$\begin{array}{c} \beta F=\beta F_{I}+\int \rho_{c}\left(r\right)\left[\ln\rho_{c}\left(r\right)-1\right]dr\\ +\int \rho_{r1}\left(r,a\right)\left[\ln\rho_{r1}\left(r,a\right)-1\right]drda+\int \rho_{r2}\left(r,a\right)\left[\ln\rho_{r2}\left(r,a\right)-1\right]drda\\ -\frac{1}{2}\int C_{cc}\left(r_{1},r_{2}\right)\delta \rho_{c}\left(r_{1}\right)\delta \rho_{c}\left(r_{2}\right)dr_{1}dr_{2}-\int C_{r1c}\left(r_{1},r_{2},a_{1}\right)\delta \rho_{r1}\left(r_{1},a_{1}\right)\delta \rho_{c}\left(r_{2}\right)dr_{1}dr_{2}da_{1}\\ -\int C_{r2c}\left(r_{1},r_{2},a_{1}\right)\delta \rho_{r2}\left(r_{1},a_{1}\right)\delta \rho_{c}\left(r_{2}\right)dr_{1}dr_{2}da_{1}\\ -\frac{1}{2}\int C_{r1r1}\left(r_{1},r_{2},a_{1},a_{2}\right)\delta \rho_{r1}\left(r_{1},a_{1}\right)\delta \rho_{r1}\left(r_{2},a_{2}\right)dr_{1}dr_{2}da_{1}da_{2}\\ -\frac{1}{2}\int C_{r2r2}\left(r_{1},r_{2},a_{1},a_{2}\right)\delta \rho_{r2}\left(r_{1},a_{1}\right)\delta \rho_{r2}\left(r_{2},a_{2}\right)dr_{1}dr_{2}da_{1}da_{2}\\ -\int C_{r1r2}\left(r_{1},r_{2},a_{1},a_{2}\right)\delta \rho_{r1}\left(r_{1},a_{1}\right)\delta \rho_{r2}\left(r_{2},a_{2}\right)dr_{1}dr_{2}da_{1}da_{2}, \end{array}$$

where $F_{I}$ is the free energy of the isotropic phase, while $C_{cc}\left(r_{1},r_{2}\right)$, $C_{ric}\left(r_{1},r_{2},a_{1}\right)$ and $C_{rirj}\left(r_{1},r_{2},a_{1},a_{2}\right)$ are the direct correlation functions of the monomers of coil and rod fragments as indicated by the corresponding indexes, and $\delta \rho_{\nu }=\rho_{\nu }-\rho_{0\nu }$ are the differences between the one-particle densities of monomers of type ν in a partially ordered phase and in the isotropic disordered phase.

Similarly to the theory of diblock copolymers, we distinguish between strong correlations of monomers within the same chain and weaker interactions between monomers in different chains. The latter can be taken into account in the so-called random phase approximation, and, for simplicity, we reduce them to a repulsion between monomers of different kinds and the Maier-Saupe orientational interactions of the rod fragments. This allows expressing the direct correlation functions as:

(7) $$C_{cc}\left(r_{1},r_{2}\right)\approx C_{cc}^{\left(I\right)}\left(r_{12}\right),$$

(8) $$C_{ric}\left(r_{1},r_{2},a_{1}\right)\approx C_{ric}^{\left(I\right)}\left(r_{12},a_{1}\right)-\chi \left(r_{12}\right)$$

(9) $$C_{r1r2}\left(r_{1},r_{2},a_{1},a_{2}\right)\approx C_{r1r2}^{\left(I\right)}\left(r_{12},a_{1},a_{2}\right)+\beta J\left(r_{12}\right)P_{2}\left(a_{1}\cdot a_{2}\right),$$

(10) $$C_{riri}\left(r_{1},r_{2},a_{1},a_{2}\right)\approx \delta \left(a_{1}-a_{2}\right)C_{riri}^{\left(I\right)}\left(r_{12},a_{1}\right)+\beta J\left(r_{12}\right)P_{2}\left(a_{1}\cdot a_{2}\right),$$

where $C_{cc}^{\left(I\right)}\left(r_{12}\right)$, $C_{ric}^{\left(I\right)}\left(r_{12},a_{1}\right)$, $C_{r1r2}^{\left(I\right)}\left(r_{12},a_{1},a_{2}\right)$, and $C_{riri}^{\left(I\right)}\left(r_{12},a_{1}\right)$ are the direct correlation functions between the monomers within the same chain in the isotropic phase of melt of noninteracting chains, and the occurrence of a delta-function in $C_{riri}$ formally reflects an infinite rigidity of each rod, as all its fragments are identically oriented. The function $\chi (r_{12})$ describes the isotropic repulsion between rod and coil monomers and the term $\beta J\left(r_{12}\right)P_{2}\left(a_{1}\cdot a_{2}\right)$ is a Maier-Saupe-like orientational interaction of all rod monomers, where $P_{2}\left(x\right)$ is the second Legendre polynomial.

Substituting these direct correlations into the free energy (6) and minimizing it with respect to $\rho_{c}\left(r\right)$ and $\rho_{ri}\left(r,a\right)$ yields the following expressions for the densities of coil and rod monomers:

(11) $$\begin{array}{c} \rho_{c}\left(r_{1}\right)=Z_{c}^{-1}\exp\left\{\int C_{cc}^{\left(I\right)}\left(r_{12}\right)\delta \rho_{c}\left(r_{2}\right)dr_{2}\right.\\ \;-\left.\int \left[\chi \left(r_{12}\right)-C_{r1c}^{\left(I\right)}\left(r_{12},a_{2}\right)\right]\delta \rho_{r1}\left(r_{2},a_{2}\right)dr_{2}da_{2}\right.\\ \;\left.-\int \left[\chi \left(r_{12}\right)-C_{r2c}^{\left(I\right)}\left(r_{12},a_{2}\right)\right]\delta \rho_{r2}\left(r_{2},a_{2}\right)dr_{2}da_{2}\right\} \end{array}$$

(12) $$\begin{array}{c} \rho_{r1}\left(r_{1},a_{1}\right)=Z_{r1}^{-1}\exp\left\{\int C_{r1r1}^{\left(I\right)}\left(r_{12},a_{1}\right)\delta \rho_{r1}\left(r_{2},a_{1}\right)dr_{2}+\right.\\ \;\left.\int C_{r1r2}^{\left(I\right)}\left(r_{12},a_{1},a_{2}\right)\delta \rho_{r2}\left(r_{2},a_{2}\right)dr_{2}da_{2}-\int \left[\chi \left(r_{12}\right)-C_{r1c}^{\left(I\right)}\left(r_{12},a_{1}\right)\right]\delta \rho_{c}\left(r_{2}\right)dr_{2}\right.\\ \;\left.+\beta \int J\left(r_{12}\right)P_{2}\left(a_{1}\cdot a_{2}\right)\left[\delta \rho_{r1}\left(r_{2},a_{2}\right)+\delta \rho_{r2}\left(r_{2},a_{2}\right)\right]dr_{2}da_{2}\right\} \end{array}$$

(13) $$\begin{array}{c} \rho_{r2}\left(r_{1},a_{1}\right)=Z_{r2}^{-1}\exp\left\{\int C_{r2r2}^{\left(I\right)}\left(r_{12},a_{1}\right)\delta \rho_{r2}\left(r_{2},a_{1}\right)dr_{2}\right.\\ \;\left.+\int C_{r1r2}^{\left(I\right)}\left(r_{12},a_{1},a_{2}\right)\delta \rho_{r1}\left(r_{2},a_{2}\right)dr_{2}da_{2}-\int \left[\chi \left(r_{12}\right)-C_{r2c}^{\left(I\right)}\left(r_{12},a_{1}\right)\right]\delta \rho_{c}\left(r_{2}\right)dr_{2}\right.\\ \;\left.+\beta \int J\left(r_{12}\right)P_{2}\left(a_{1}\cdot a_{2}\right)\left[\delta \rho_{r1}\left(r_{2},a_{2}\right)+\delta \rho_{r2}\left(r_{2},a_{2}\right)\right]dr_{2}da_{2}\right\} \end{array}$$

where $Z_{c}$, $Z_{r1}$, and $Z_{r2}$ are the corresponding normalization factors.

### 2.2. Free Energy of the Lamellar Phase

In the lamellar phase, all one-particle densities are periodic functions of the position along the axis of the phase and have the same period. Thus one concludes that the effective mean-field potentials are also periodic and one can expand them in Fourier series taking into account the first dominant harmonics. By appropriately choosing the coordinate origin, the mean-field potentials can be considered as even functions of the position, and the corresponding Fourier expansions contain only cosine terms.

The details of the Fourier expansion of all terms in Equations (11–13) are presented in Appendix A. In particular, the coefficients of such an expansion are proportional to the Fourier transforms of the direct correlation functions or to the functions χ(q) and J(q). Some of these coefficients, which depend on the orientation of the rods, can also be expanded in Legendre polynomials taking into account the first few terms which enables one to express the densities and the free energy as a function of the orientational and translational order parameters.

For example, the Fourier transform of the coil-coil direct correlation function depends only on the magnitude of the wave vector q, i.e., $C_{cc}=C_{cc}\left(q\right)$. In contrast, the direct correlation function between rod and coil monomers depends on the orientation of the rod and can be approximated as:

(14) $$C_{ric}^{\left(I\right)}\left(q,a\right)\approx C_{ric}^{\left(0\right)}\left(q\right)+C_{ric}^{\left(2\right)}\left(q\right)P_{2}\left(a\cdot k\right),$$

where the unit vector k is along q.

The direct correlation function for the two segments of the same rod can be approximately expressed in a similar way:

(15) $$C_{riri}^{\left(I\right)}\left(q,a_{1}\right)\approx 4\pi C_{riri}^{\left(0\right)}\left(q\right)+4\pi C_{riri}^{\left(2\right)}\left(q\right)P_{2}\left(a_{1}\cdot k\right).$$

Finally, the direct correlation function between the segments of the two different rods depends on the orientations $a_{1}$ and $a_{2}$ of both rods:

(16) $$C_{r1r2}^{\left(I\right)}\left(q,a_{1},a_{2}\right)\approx C_{r1r2}^{\left(0\right)}\left(q\right)+C_{r1r2}^{\left(2\right)}\left(q\right)\left[P_{2}\left(a_{1}\cdot k\right)+P_{2}\left(a_{2}\cdot k\right)\right]+C_{r1r2}^{\left(3\right)}\left(q\right)P_{2}\left(a_{1}\cdot a_{2}\right),$$

As shown in Appendix A, the free energy of the lamellar phase in triblock rod-coil-rod copolymers can finally be expressed in the following approximate form:

(17) $$\begin{array}{c} \beta F/V=\rho_{0}^{2}\left[f_{r1}^{2}C_{r1r1}^{\left(2\right)}\left(q\right)\psi_{r1}\sigma_{1}+f_{r2}^{2}C_{r2r2}^{\left(2\right)}\left(q\right)\psi_{r2}\sigma_{2}+f_{r1}f_{r2}C_{r1r2}^{\left(2\right)}\left(q\right)\left(\psi_{r1}\sigma_{2}+\psi_{r2}\sigma_{1}\right)\right.\\ \;\left.+f_{c}f_{r1}C_{r1c}^{\left(2\right)}\left(q\right)\psi_{c}\sigma_{1}+f_{c}f_{r2}C_{r2c}^{\left(2\right)}\left(q\right)\psi_{c}\sigma_{2}\right]\\ \;+\rho_{0}^{2}C_{r1r2}^{\left(4\right)}f_{r1}f_{r2}S_{1}S_{2}+\frac{1}{2}\rho_{0}^{2}\beta J_{0}{\left(S_{1}f_{r1}+S_{2}f_{r2}\right)}^{2}\\ \;+\rho_{0}^{2}\left[\beta J_{2}+C_{r1r2}^{\left(3\right)}\left(q\right)\right]f_{r1}f_{r2}\sigma_{1}\sigma_{2}+\frac{1}{2}\rho_{0}^{2}\left[\beta J_{2}+5C_{r1r1}^{\left(0\right)}\left(q\right)+\frac{10}{7}C_{r1r1}^{\left(2\right)}\left(q\right)\right]\sigma_{1}^{2}f_{r1}^{2}\\ \;+\frac{1}{2}\rho_{0}^{2}\left[\beta J_{2}+5C_{r2r2}^{\left(0\right)}\left(q\right)+\frac{10}{7}C_{r2r2}^{\left(2\right)}\left(q\right)\right]\sigma_{2}^{2}f_{r2}^{2}\\ \;+\frac{1}{2}\rho_{0}^{2}f_{c}^{2}C_{cc}^{\left(0\right)}\left(q\right)\psi_{c}^{2}+\frac{1}{2}\rho_{0}^{2}f_{r1}^{2}C_{r1r1}^{\left(0\right)}\left(q\right)\psi_{r1}^{2}+\frac{1}{2}\rho_{0}^{2}f_{r2}^{2}C_{r2r2}^{\left(0\right)}\left(q\right)\psi_{r2}^{2}+\rho_{0}^{2}f_{r1}f_{r2}C_{r1r2}^{\left(0\right)}\left(q\right)\psi_{r1}\psi_{r2}\\ \;+\rho_{0}^{2}f_{c}f_{r1}C_{r1c}^{\left(0\right)}\left(q\right)\psi_{r1}\psi_{c}+\rho_{0}^{2}f_{c}f_{r2}C_{r2c}^{\left(0\right)}\left(q\right)\psi_{r2}\psi_{c}-\rho_{0}^{2}f_{r1}f_{c}\chi \psi_{c}\psi_{r1}-\rho_{0}^{2}f_{r2}f_{c}\chi \psi_{c}\psi_{r2}\\ \;-\rho_{0}f_{r1}\ln Z_{r1}-\rho_{0}f_{c}\ln Z_{c}-\rho_{0}f_{r2}\ln Z_{r2} \end{array}$$

where *V* is the polymer volume and the partition functions read as

(18) $$\begin{array}{c} Z_{c}=\int dz\exp\left[\rho_{0}\cos\left(qz\right)\left(f_{c}C_{cc}^{\left(0\right)}\left(q\right)\psi_{c}+\;f_{r1}C_{r1c}^{\left(2\right)}\left(q\right)\sigma_{1}+f_{r1}C_{r1c}^{\left(0\right)}\left(q\right)\psi_{r1}+\right.\right.\\ \;\left.\left.\;f_{r2}C_{r2c}^{\left(2\right)}\left(q\right)\sigma_{2}+f_{r2}C_{r2c}^{\left(0\right)}\left(q\right)\psi_{r2}-f_{r1}\chi \psi_{r1}-f_{r2}\chi \psi_{r2}-\lambda \right)\right], \end{array}$$

(19) $$\begin{array}{c} Z_{r1}=\int dzda\exp\left[\rho_{0}\cos\left(qz\right)\left(f_{r1}C_{r1r1}^{\left(0\right)}\left(q\right)\psi_{r1}+f_{r2}C_{r1r2}^{\left(0\right)}\left(q\right)\psi_{r2}+f_{c}C_{r1c}^{\left(0\right)}\left(q\right)\psi_{c}-f_{c}\chi \psi_{c}-\lambda \right)\right.\\ \;+\rho_{0}\cos\left(qz\right)P_{2}\left(a\cdot k\right)\left(f_{c}C_{r1c}^{\left(2\right)}\left(q\right)\psi_{c}+\frac{1}{4\pi }f_{r1}C_{r1r1}^{\left(2\right)}\left(q\right)\psi_{r1}+f_{r2}C_{r1r2}^{\left(2\right)}\left(q\right)\psi_{r2}\right.\\ \;\left.+f_{r2}\left[\beta J_{2}+C_{r1r2}^{\left(3\right)}\left(q\right)\right]\sigma_{2}+f_{r1}\left[\beta J_{2}+5C_{r1r1}^{\left(0\right)}\left(q\right)+\frac{10}{7}C_{r1r1}^{\left(2\right)}\left(q\right)\right]\sigma_{1}\right)\\ \;+\rho_{0}\cos\left(qz\right)\left[f_{r1}C_{r1r1}^{\left(2\right)}\left(q\right)\sigma_{1}+f_{r2}C_{r1r2}^{\left(2\right)}\left(q\right)\sigma_{2}\right]\\ \left.\;+\rho_{0}\beta J_{0}\left(f_{r1}S_{1}+f_{r2}S_{2}\right)P_{2}\left(a\cdot k\right)+\rho_{0}f_{r2}C_{r1r2}^{\left(4\right)}S_{2}P_{2}\left(a\cdot k\right)\right), \end{array}$$

(20) $$\begin{array}{c} Z_{r2}=\int dzda\exp\left[\rho_{0}\cos\left(qz\right)\left(f_{r2}C_{r2r2}^{\left(0\right)}\left(q\right)\psi_{r2}+f_{r1}C_{r1r2}^{\left(0\right)}\left(q\right)\psi_{r1}+f_{c}C_{r2c}^{\left(0\right)}\left(q\right)\psi_{c}-f_{c}\chi \psi_{c}-\lambda \right)\right.\\ \;+\rho_{0}\cos\left(qz\right)P_{2}\left(a\cdot k\right)\left(f_{c}C_{r2c}^{\left(2\right)}\left(q\right)\psi_{c}+f_{r2}\frac{1}{4\pi }C_{r2r2}^{\left(2\right)}\left(q\right)\psi_{r2}+f_{r1}C_{r1r2}^{\left(2\right)}\left(q\right)\psi_{r1}\right.\\ \;\left.+f_{r1}\left[\beta J_{2}+C_{r1r2}^{\left(3\right)}\left(q\right)\right]\sigma_{1}+f_{r2}\left[\beta J_{2}+5C_{r2r2}^{\left(0\right)}\left(q\right)+\frac{10}{7}C_{r2r2}^{\left(2\right)}\left(q\right)\right]\sigma_{2}\right)\\ \;+\cos\left(qz\right)\rho_{0}\left[f_{r2}C_{r2r2}^{\left(2\right)}\left(q\right)\sigma_{2}+f_{r1}C_{r1r2}^{\left(2\right)}\left(q\right)\sigma_{1}\right]\\ \;\left.+\rho_{0}\beta J_{0}\left(f_{r1}S_{1}+f_{r2}S_{2}\right)P_{2}\left(a\cdot k\right)+\rho_{0}f_{r1}C_{r1r2}^{\left(4\right)}S_{1}P_{2}\left(a\cdot k\right)\right), \end{array}$$

where the *z*-axis points along the wavevector q. Definitions of all coefficients are given in Appendix A, and $S_{i}$, $\psi_{i}$ and $\sigma_{i}$ are the order parameters of the fragments of rods of type *i* in the lamellar phase. In particular, the nematic order parameters, $S_{1}$ and $S_{2}$, are conventionally introduced as

(21) $$S_{i}={\langle P_{2}\left(a\cdot k\right)\rangle }_{ri}=\int f_{1}^{\left(ri\right)}\left(r,a\right)P_{2}\left(a\cdot k\right)drda,$$

where i=1,2. The mixed order parameters

(22) $$\sigma_{i}={\langle P_{2}\left(a\cdot k\right)\cos\left(q\cdot r\right)\rangle }_{ri}=\int f_{1}^{\left(ri\right)}\left(r,a\right)P_{2}\left(a\cdot k\right)\cos\left(q\cdot r\right)drda,$$

characterize the degree of simultaneous orientational and positional ordering of the rods of type *i*.

In the expression for both order parameters, the averaging ${\langle \cdots \rangle }_{ri}$ is performed with the single-particle distribution function $f_{1}^{\left(ri\right)}\left(r,a\right)$ of the fragments of rods of type *i*, which is related to the corresponding density as $\rho_{ri}\left(r,a\right)=N_{ri}f_{1}^{\left(ri\right)}\left(r,a\right)=V\rho_{0}f_{ri}f_{1}^{\left(ri\right)}\left(r,a\right)$, where $\rho_{0}$ is the density of the fragments of all types, $N_{ri}$ is the total number of the corresponding rod fragments and $f_{ri}$ is their relative fraction. Note that the density differences entering Equations (11)–(13) are defined as $\delta \rho_{ri}\left(r,a\right)=\rho_{ri}\left(r,a\right)-\frac{1}{4\pi }\rho_{0}f_{ri}$ but their latter constant parts provide vanishing contributions to the integrals.

Finally, the positional order parameter of the coil segments is defined by the equation:

(23) $$\psi_{c}={\langle \cos(q\cdot r)\rangle }_{c}=\int f_{1}^{\left(c\right)}\left(r\right)\cos\left(q\cdot r\right)dr,$$

while the positional order parameter for the rod segments is expressed as:

(24) $$\psi_{ri}={\langle \cos(q\cdot r)\rangle }_{ri}=\int f_{1}^{\left(ri\right)}\left(r,a\right)\cos\left(q\cdot r\right)drda.$$

The incompressibility condition requires the order parameters (23) and (24) to obey the equation

(25) $$f_{c}\psi_{c}+f_{r1}\psi_{r1}+f_{r2}\psi_{r2}=0,$$

which can be ensured by adding to the free energy a corresponding term with a Lagrangian multiplier λ. The free energy can then be minimized with respect to the order parameters and the Lagrange multiplier λ to obtain a conditional minimum with the relation (25) being precisely fulfilled. In practice, such a minimization can be performed only if the direct correlation functions of the reference system of noninteracting triblock copolymer molecules are known. These correlation functions are calculated below.

## 3. Correlation Functions in Rod-Coil-Rod Triblock Copolymers

### 3.1. Ornstein-Zernike Equations

Direct pair correlation functions of block copolymers are not known explicitly but they are generally related to the corresponding total pair correlation functions by the Ornstein-Zernike equations. The total correlation functions, in turn, can readily be expressed in terms of the density-density correlation functions which in principle can be evaluated for any combination of Gaussian chains and rigid rods.

In the case of a simple isotropic fluid composed of spherical molecules, the Ornstein-Zernike equation can be written in the form

(26) $$h\left(r_{12}\right)=C\left(r_{12}\right)+\rho_{0}\int C\left(r_{13}\right)h\left(r_{23}\right)dr_{3},$$

which can readily be solved in the Fourier representation. Here the first term describes the direct correlation between molecules “1” and “2” while the second term corresponds to the indirect correlation via the third particle “3”. In the case of multi-component fluids composed of anisotropic particles, including rod-coil copolymers, the Ornstein-Zernike equations are significantly more complicated because they include several terms describing indirect correlations and these terms may involve integration over all orientations of the third particle. Thus the Ornstein-Zernike equations for rod-coil-rod triblock copolymers are nontrivial, and they have never been presented in the literature. The detailed derivation of the Ornstein-Zernike equations for rod-coil-rod triblock copolymers is described in Appendix B, where it is shown that there are six independent Ornstein-Zernike equations.

Firstly, both the direct and the total pair correlation between coil monomers in the isotropic phase depend only on the distance between the segments and hence the corresponding Ornstein-Zernike equation can be written in the form:

(27) $$\begin{array}{c} h_{cc}\left(r\right)=C_{cc}^{\left(I\right)}\left(r\right)+\frac{1}{4\pi }\rho_{0}f_{r1}\int C_{r1c}^{\left(I\right)}\left(r-r^{\prime },a_{1}\right)h_{r1c}\left(r^{\prime },a_{1}\right)dr^{\prime }da_{1}\\ \;+\frac{1}{4\pi }\rho_{0}f_{r2}\int C_{r2c}^{\left(I\right)}\left(r-r^{\prime },a_{2}\right)h_{r2c}\left(r^{\prime },a_{2}\right)dr^{\prime }da_{2}+\rho_{0}f_{c}\int C_{cc}^{\left(I\right)}(|r-r^{\prime }\left|\right)h_{cc}\left(r^{\prime }\right)dr^{\prime }, \end{array}$$

In contrast, the correlation functions between coil segments and those of the rod 1 depend also on the unit vector $a_{1}$ along the rod 1. Then the corresponding Ornstein-Zernike equation is expressed as:

(28) $$\begin{array}{c} h_{r1c}\left(r,a_{1}\right)=C_{r1c}^{\left(I\right)}\left(r,a_{1}\right)+\frac{1}{4\pi }\rho_{0}f_{r1}\int h_{r1r1}\left(r^{\prime },a_{1}\right)C_{r1c}^{\left(I\right)}\left(r-r^{\prime },a_{1}\right)dr^{\prime }\\ \;+\frac{1}{4\pi }\rho_{0}f_{r2}\int h_{r1r2}\left(r^{\prime },a_{1},a_{2}\right)C_{r2c}^{\left(I\right)}\left(r-r^{\prime },a_{2}\right)dr^{\prime }da_{2}+\rho_{0}f_{c}\int h_{r1c}\left(r^{\prime },a_{1}\right)C_{cc}^{\left(I\right)}(|r-r^{\prime }\left|\right)dr^{\prime }, \end{array}$$

Note that the orientation of all segments of the rod 1 is the same and hence their density does not include a factor of ${\left(4\pi \right)}^{-1}$. The Ornstein-Zernike equation for the correlation functions of coil segments with those of the rod 2 can be obtained by swapping the indices “1”and “2” in Equation (28).

The pair correlation functions between different segments of the same rod i=1,2 depend on the same orientation $a_{i}$ of this rod and, therefore, the corresponding Ornstein-Zernike equations reads as:

(29) $$\begin{array}{c} h_{r1r1}\left(r,a_{1}\right)=C_{r1r1}^{\left(I\right)}\left(r,a_{1}\right)+\frac{1}{4\pi }\rho_{0}f_{r1}\int C_{r1r1}^{\left(I\right)}\left(r-r^{\prime },a_{1}\right)h_{r1r1}\left(r^{\prime },a_{1}\right)dr^{\prime }\\ \;+\frac{1}{4\pi }\rho_{0}f_{r2}\int C_{r1r2}^{\left(I\right)}\left(r-r^{\prime },a_{1},a_{2}\right)h_{r1r2}\left(r^{\prime },a_{1},a_{2}\right)dr^{\prime }da_{2}\\ \;+\rho_{0}f_{c}\int C_{r1c}^{\left(I\right)}\left(r-r^{\prime },a_{1}\right)h_{r1c}\left(r^{\prime },a_{1}\right)dr^{\prime }, \end{array}$$

The corresponding equation for $h_{r2r2}\left(r,a_{1}\right)$ can again be obtained by swapping the indices “1” and “2” .

The remaining Ornstein-Zernike equation for the correlations between segments of different rods depends on the orientations of both rods and can be written in the following form:

(30) $$\begin{array}{c} h_{r1r2}\left(r,a_{1},a_{2}\right)=C_{r1r2}^{\left(I\right)}\left(r,a_{1},a_{2}\right)+\frac{1}{4\pi }\rho_{0}f_{r1}\int h_{r1r1}\left(r^{\prime },a_{1}\right)C_{r1r2}^{\left(I\right)}\left(r-r^{\prime },a_{1},a_{2}\right)dr^{\prime }\\ \;+\frac{1}{4\pi }\rho_{0}f_{r2}\int h_{r1r2}\left(r^{\prime },a_{1},a_{2}\right)C_{r2r2}^{\left(I\right)}\left(r-r^{\prime },a_{2}\right)dr^{\prime }+\rho_{0}f_{c}\int h_{r1c}\left(r^{\prime },a_{1}\right)C_{r2c}^{\left(I\right)}\left(r-r^{\prime },a_{2}\right)dr^{\prime }. \end{array}$$

The Ornstein-Zernike equations make a set of coupled integral equations which cannot be solved analytically. However, it is possible to obtain an approximate solution by using the expansions of the direct correlation functions given by Equations (14)–(16) and expanding the total correlation functions in a similar way. As shown in Appendix C, one obtains a system of 12 linear equation for the expansion coefficients of the direct correlation functions which enter the expression for the free energy of the lamellar phase. These equations can readily be solved numerically and the results can then be substituted into the free energy. One notes that this procedure requires the knowledge of the total pair correlation functions of rod-coil-rod triblock copolymers which are presented in the following section.

### 3.2. Density-Density Correlation Functions of Rod-Coil-Rod Triblock Copolymers

The coil-coil density correlation function for Gaussian chains has been calculated by many authors (see e.g., Ref. [61]) and can be written in the form:

(31) $$h_{cc}\left(q\right)=\frac{4N}{\rho_{0}f_{c}^{2}}\frac{1}{x^{2}}\left[f_{c}x+\exp\left(-f_{c}x\right)-1\right].$$

where $f_{c}$ is the fraction of coil segments and $x=q^{2}Na^{2}/6=q^{2}R^{2}$.

Total pair correlation functions between rod and coil monomers and between monomers of the same rod have been used in the theory of rod-coil diblock copolymers [46] without providing a detailed derivation. We derive the correlation functions used in Ref. [46] in Appendix D and also calculate there for the first time to our knowledge those arising in the triblock molecules. In particular, as shown in Appendix D, the Fourier transform of the rod-coil total pair correlation function is expressed as:

(32) $$h_{ric}\left(q,a\right)=2\int h_{ric}\left(r,a\right)\cos\left(q\cdot r\right)dr=\frac{2N}{\rho_{0}f_{c}}\frac{1}{x}\left[1-\exp(-f_{c}x)\right]\frac{\sin y_{i}}{y_{i}},$$

where $y_{i}=Nf_{ri}a\left(q\cdot a\right)$. The cosine Fourier transform of the rod-rod total correlation functions between the segments of the same rod reads:

(33) $$\begin{array}{c} h_{riri}\left(q,a\right)=2\int h_{riri}\left(r,a\right)\cos\left(q\cdot r\right)dr=\frac{8\pi }{\rho_{0}f_{ri}^{2}}\frac{1}{N}\sum_{j\neq k}^{Nf_{ri}}\cos\left[\left(k-j\right)a\left(q\cdot a\right)\right]\\ \;\approx \frac{8\pi }{\rho_{0}f_{ri}^{2}}\frac{1}{N}\int_{0}^{Nf_{ri}}\int_{0}^{Nf_{ri}}\cos\left[\left(k-j\right)a\left(q\cdot a\right)\right]dk\;dj=16\pi \frac{N}{\rho_{0}}\frac{1-\cos y_{i}}{y_{i}^{2}}. \end{array}$$

Finally, the total correlation function for the monomers of different rods separated by a coil has not been presented in the literature. As shown in Appendix D, its cosine Fourier transform can be written in the form:

(34) $$\begin{array}{c} h_{r1r2}\left(q,a_{1},a_{2}\right)=2\int h_{r1r2}\left(r,a_{1},a_{2}\right)\cos\left(q\cdot r\right)dr=\frac{2}{\rho_{0}f_{r1}f_{r2}}\frac{1}{N}\sum_{j=1}^{Nf_{r1}}\sum_{k=1}^{Nf_{r2}}P_{jk}\left(q,a_{1},a_{2}\right)\\ \;\approx \frac{2}{\rho_{0}f_{r1}f_{r2}}\frac{\exp(-f_{c}x)}{N}\int_{0}^{Nf_{r1}}\int_{0}^{Nf_{r2}}\cos\left[a\left(ja_{1}-ka_{2}\right)\cdot q\right]dkdj\\ =\frac{2}{\rho_{0}}N\frac{\exp(-f_{c}x)}{y_{1}y_{2}}\left[\cos\left(y_{1}-y_{2}\right)-\cos y_{1}-\cos y_{2}+1\right]. \end{array}$$

## 4. Results and Discussion

### 4.1. Phase Diagrams and Transitions

We employ MATLAB fminsearch routine to minimize the free-energy density (17) as a function of all the order parameters. Note that the nematic order parameters of rod monomers enter in Equation (17) only via a combination $\left(f_{r1}S_{1}+f_{r2}S_{2}\right)$, which we use as one of the minimization variables. The nematic phase corresponds to a free-energy minimum where only this parameter is nonzero.

In the lamellar phase, all the introduced order parameters are nonzero, and the phase wavenumber *q* should be also considered as a minimization variable. We organize the corresponding numerical procedure in three stages. Firstly, the system of linear equations for the expansion coefficients of the direct correlation functions, presented in Appendix C, is solved numerically and the results are substituted in the expression for the free energy. At the second stage, for each guessed value of the wavenumber *q*, the local minimum is found and the corresponding value of the free energy is evaluated. Finally, we vary the wavenumber *q* to compare the energy in all such local minima and to identify the global free-energy minimum. On each step, we verify that the incompressibility and the self-consistence conditions are accurately fulfilled. The decision on the stable phase for particular sets of parameters is taken by comparing the free energy values of the disordered state (zero energy), the nematic state, and the lamellar state.

For convenience, we use the dimensionless temperature $\tau =k_{B}T/J_{0}$ determined by the parameter $J_{0}$ of the main Maier-Saupe-like term in Equation (A4). As the interactions of rod monomers are supposed to be short-ranged compared to the rod length and the lamellar periodicity, we set the other parameter to $J_{2}=2J_{0}$, which corresponds to the limit q→0 in Equation (A5).

**Figure 1:**
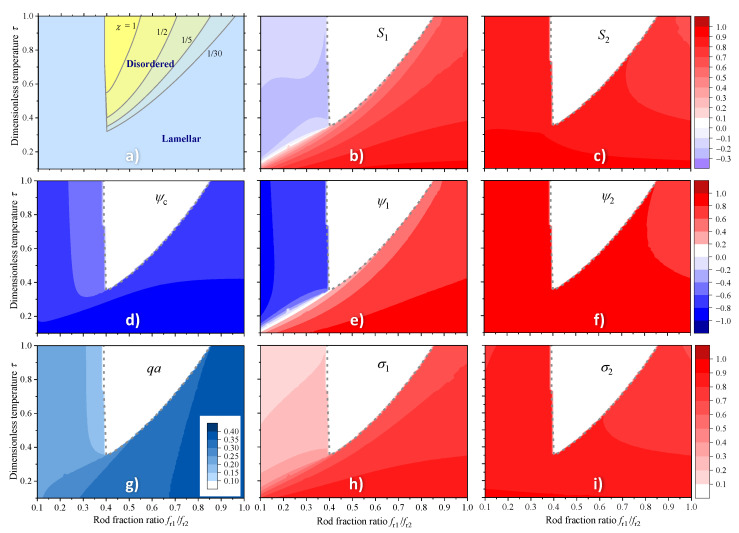
Phase diagrams, order parameters and equilibrium wavenumber in the axes of rod fraction ratio $f_{r1}/f_{r2}$—dimensionless temperature $\tau =k_{B}T/J_{0}$ of asymmetric rod-coil-rod triblock copolymer calculated numerically by minimizing the free energy (17) for N=30, $J_{2}=2J_{0}$ and $f_{c}=0.5$. Phase diagrams for different indicated values of the Flory-Huggins parameter χ are shown in (**a**). The colormaps (**b**–**i**) are calculated for χ=1/5 and represent the variation of orientational (**b**,**c**), translational (**d**–**f**) and mixed (**h**,**i**) order parameters in the lamellar phase. The colormap in (**g**) shows the wavenumber of the lamellar structure.

A set of typical phase diagrams for asymmetric rod-coil-rod triblock copolymers in terms of the rod fraction ratio $f_{r1}/f_{r2}$ — dimensionless temperature τ is combined in Figure 1a. One notes that the lamellar phase is less stable in the region $f_{r1}/f_{r2}\approx$ 0.4–0.5 where the corner-shaped region of the disordered phase is observed on the phase diagram. From this region, the transition into the lamellar phase occurs both with the increasing and with the decreasing asymmetry of the rods. In particular, for $f_{r1}/f_{r2}>0.5$ the transition temperature into the disordered phase gradually increases with the decreasing asymmetry of the rods. The lamellar phase is also stable in the region $f_{r1}/f_{r2}<0.4$ but it should be noted that its structure in this domain is very different. Indeed, for $f_{r1}/f_{r2}>0.4$ the transition into the disordered phase is strongly temperature dependent which indicates that both phase separation effects, controlled by the Flory-Huggins parameter χ, and the periodic term in the mean filed potential, controlled by the Mayer-Saupe orientational interaction parameter $J_{2}$, promote the lamellar ordering. For $f_{r1}/f_{r2}<0.4$, on the contrary, the boundary between the lamellar and the disordered phase is practically temperature independent which indicates that the corresponding transition is governed by athermal phase separation effects.

Drastic difference in the structure of lamellar phase in the region $f_{r1}/f_{r2}<0.4$ and for $f_{r1}/f_{r2}>0.5$ becomes obvious from the analysis of the order parameter colormaps shown in Figure 1b–f,h,i. One can readily see that for $f_{r1}/f_{r2}>0.5$, the distribution of all order parameters of rods of both types is qualitatively the same, although certain quantitative differences remain determined by different rod lengths. All order parameters are close to unity and do not vary significantly. Thus the triblock copolymer is strongly ordered both translationally and orientationally in this region.

In contrast, for $f_{r1}/f_{r2}<0.4$, the behavior of the order parameters is qualitatively different. In particular, both orientational and translational order parameters of shorter rods are much smaller than those of the longer rods, and they even turn negative at higher temperatures. Considering the behavior of the orientational order parameters, we unambiguously point out that for $f_{r1}/f_{r2}<0.4$ the short rods remain strongly disordered and even have a weak tendency to align parallel to the lamellar planes, while the long rods remain strongly ordered along the lamella normal. The peculiar values of the translational order parameters indicate that while longer rods remain well separated from coils (in a same way as they are in diblock copolymers), the shorter rods tend to arrange layers within the coil fragments effectively avoiding their longer counterparts. Remarkably, the shorter rods separate from the longer ones despite the fact that the segments of both rods are equivalent and attract each other. Avoiding further speculations about the mechanism of such microphase separation, we assume that it is determined by entropic effects associated with large difference in the rod lengths.

**Figure 2:**
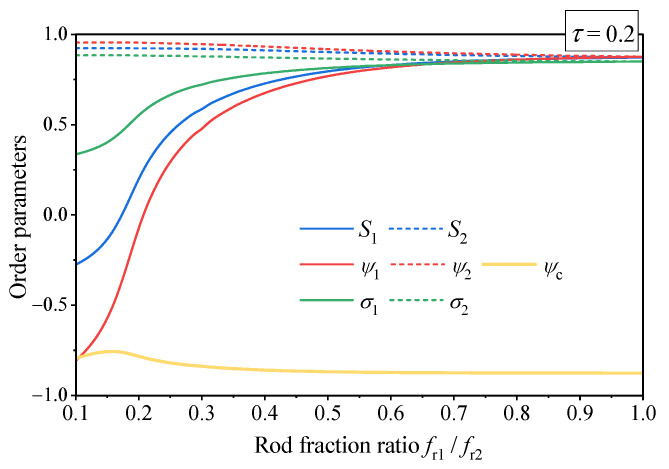
Order parameters of lamellar phase of the asymmetric rod-coil-rod triblock copolymer as functions of the rod fraction ratio $f_{r1}/f_{r2}$ at a constant dimensionless temperature τ=0.2. All other model parameters are the same as in Figure 1.

In spite of qualitatively different lamellar ordering at low and high triblock asymmetry, we do not observe a distinct phase transition, which could hypothetically exist between different lamellar phases. Instead, as is exemplified in Figure 2, with growing asymmetry (decreasing rod fraction ratio) the copolymer gradually transforms from a state with all order parameters close to unity to a state with orientationally disordered shorter rods which are spatially distributed together with the coil fragments, i.e., effectively avoiding their longer counterparts.

**Figure 3:**
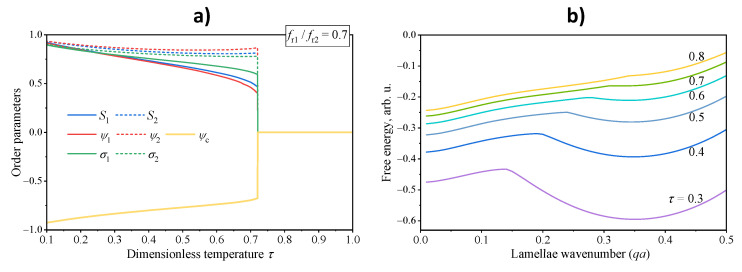
Transition from lamellar to disordered phase of the asymmetric rod-coil-rod triblock copolymer with $f_{r1}/f_{r2}=0.7$ with growing dimensionless temperature τ. (**a**) Temperature dependencies of all order parameters. (**b**) Dependencies of free energy density on the lamellae wavenumber at different temperature values indicated on the lines. All other model parameters are the same as in Figure 1.

The transition from the lamellar to disordered state with growing temperature, on the contrary, is remarkably abrupt. Typical temperature variation of the order parameters shown in Figure 3a illustrate that this is a strong first order phase transition. The inherent nature of the transition appears to be though more peculiar than one can expect from the analogies with low molecular weight liquid crystals. As we evaluate the minimum free energy by consequent minimizations with respect to the order parameters and the wavenumber, it is possible for us to reveal how the latter affects the free energy value. We present in Figure 3b a typical set of such dependencies at several temperatures below and above the phase transition. One can readily see that below the transition temperature, the free energy is negative and exhibits a distinct minimum at a certain wavenumber. Closer to the transition, this minimum on the free energy elevates and eventually the lamellar state with a finite wavenumber becomes less favorable compared to a homogeneous state with zero wavenumber. Above the transition, the former minimum disappears and the system can attain only the homogeneous state. Note that in all cases the free energy remains negative, i.e., for an arbitrary wavenumber the lamellar state remains more favorable than a fully disordered phase having zero energy. Although the polymer on its own is in a frustrated state here, a weak external influence can provide a stable or, at least, a meta-stable lamellar structure with a desired wavenumber.

### 4.2. Computer Simulations of Lamellar Phase

DPD is nowadays considered as one of the most powerful mesoscopic methods for the simulations of self-assembling in block copolymers. Large integration step in the equations of motion and low friction coefficient for the particles with soft potentials allow one to address macromolecular ordering on large space-time scales. Previously, we used DPD to check the predictions of the molecular theory of rod-coil diblock copolymers regarding the possibility of the tilting transition [45] and to evaluate the parameters that characterize the tilted lamellar phase [57]. Note that the DPD method was originally developed for flexible polymers [62] and it needs modification when one of the copolymer blocks is stiff. To this end, we treated such blocks as rigid bodies using an algorithm by Miller et al. [63]. Here we used the same approach for the simulation of rod-coil-rod triblock copolymers. More technical details can be found in our previous papers [45,64]. The calculations were performed using a free source code LAMMPS [65] in a periodic simulation box of the size $l_{x}\times l_{y}\times l_{z}=\left(32\times 32\times 32\right)r_{c}^{3}$ (where $r_{c}$ is a certain cut-off radius that is treated as a unit length) filled with a total of 98,304 DPD particles.

**Figure 4:**
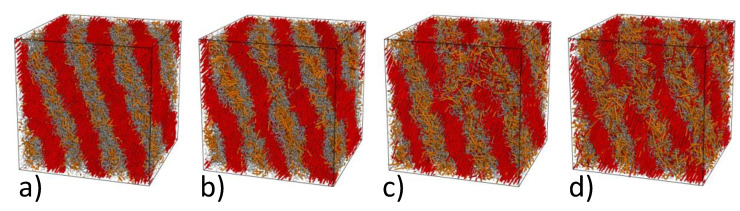
Snapshots of the triblock $A_{10}B_{10}A_{x}$ rod-coil-rod (red-gray-orange) copolymers at weak immiscibility between A and B particles ($a_{AB}=30,a_{AA}=a_{BB}=25$) for x=3 (**a**), x=4 (**b**), x=5 (**c**), and x=6 (**d**).

First of all, we prepared a microphase separated state in the symmetric $A_{10}B_{10}$ diblock copolymer, where *A* is a rigid block and *B* is a flexible block. For a rod-coil copolymer, rather weak repulsion between chemically different particles ($a_{AB}=30,a_{AA}=a_{BB}=25$) was enough to attain a well-defined lamellar morphology after $4\cdot 10^{6}$ time steps. Rigid blocks are locally ordered within lamellae, which reflects a strong tendency of rods to stacking provided their length exceeds 7 units, which agrees with our previous simulations [45,64]. Further on, we attached a shorter rigid $A_{x}$ (x=3,…,6) block to the free end of every *B* block thus obtaining a $A_{10}B_{10}A_{x}$ triblock copolymer (some of the diblocks were discarded during this process to maintain the constant density ρ=3 in the simulation cell) and equilibrated the system for $2\cdot 10^{6}$ time steps. The resulting morphologies are shown in Figure 4.

One can see that shorter rigid *A* blocks shown with orange color prefer to be mixed with flexible *B* blocks (gray) rather than with chemically identical longer *A* blocks (red). The tendency of longer rigid blocks to stacking overcomes the effective repulsion between *A* and *B* units and the lamellar morphology persists for all the situations illustrated in Figure 4. The lamellae remain nearly perfect for the shortest third block (x=3, Figure 4a), whereas its elongation leads to undulations (x=4, Figure 4b) and more substantial smearing defects (x=5 and 6, Figure 4c,d). Note that an increase in the $A_{x}$ length not only reduces the disparity between the lengths of the rigid blocks but also makes the copolymer compositionally more asymmetric, which, in turn, can destabilize the lamellar order.

**Figure 5:**
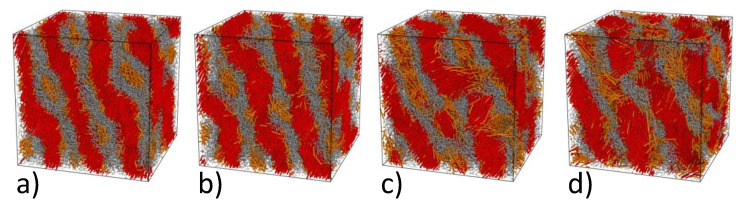
Snapshots of the triblock $A_{10}B_{10}A_{x}$ rod-coil-rod (red-gray-orange) copolymers at stronger immiscibility between A and B particles ($a_{AB}=50,a_{AA}=a_{BB}=25$) for x=3 (**a**), x=4 (**b**), x=5 (**c**), and x=6 (**d**).

**Figure 6:**
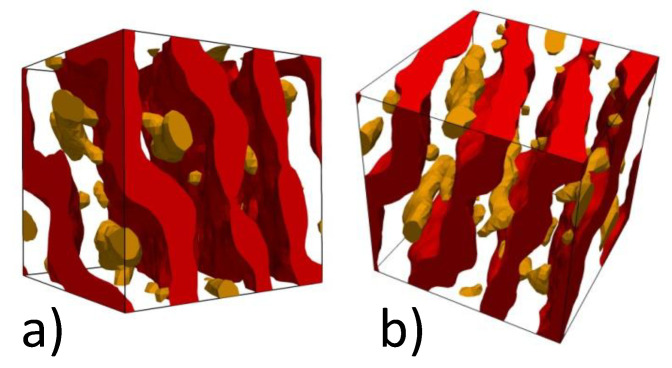
Morphology of the domains formed by longer (red) and shorter (brown) rigid A blocks of the $A_{10}B_{10}A_{4}$ copolymer for $a_{AB}=50$, $a_{AA}=a_{BB}=25$. Snapshots (**a**,**b**) are taken from different viewpoints.

After that we gradually increased the repulsion between A and B particles in the $A_{10}B_{10}A_{x}$ copolymers by steps of $\Delta a_{AB}=1$ up to $a_{AB}=50$ (which corresponds to the Flory-Huggins parameter χ=7.65) with a relaxation for $2.5\cdot 10^{5}$ time steps after each stage. The resulting morphologies are shown in Figure 5. Growing immiscibility leads to a separation between shorter rigid $A_{x}$ blocks and flexible $B_{10}$ blocks. The rigid blocks can either form micelles in the *B* domains or stick to the existing *A* lamellae formed by longer rigid $A_{10}$ blocks. The micelles, which correspond to a more extended conformation of the triblock copolymer chains, dominate at x=3 and 4 (Figure 5a,b). Their presence causes a periodic modulation in the thicknesses of lamellar A and B domains. Morphology of the domains formed by longer and shorter rigid A blocks at x=4 is shown in Figure 6. It is clear that $A_{4}$ blocks indeed form cylindrical micelles and each micelle has a few contacts with a neighboring lamellar $A_{10}$ domain, i.e., all A domains in the system are interconnected.

At the same time, even in Figure 5a,b and Figure 6 some of the $A_{x}$ blocks are tightly adsorbed at the interface between the lamellar A and B layers and this trend strongly increases at x=5 and 6 (Figure 5c,d). Since $A_{5}$ and $A_{6}$ blocks, which are still too short for stacking, occupy a considerable part of the system volume, they markedly decrease the degree of ordering in the simulated system.

**Figure 7:**
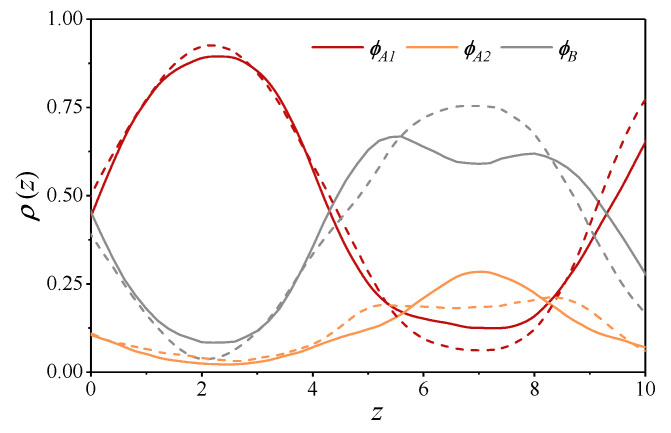
Lamellar ordering in the $A_{10}B_{10}A_{4}$ triblock copolymer: the profiles of the component volume fractions, $\phi_{A1}\left(z\right)$, $\phi_{A2}\left(z\right)$, and $\phi_{B}\left(z\right)$ calculated for the two values of the Flory-Huggins parameter χ=1.53 (solid lines) and χ=6.12 (dashed lines).

Figure 7 presents the spatial distribution of the component volume fractions, $\phi_{A1}$ (longer rigid blocks), $\phi_{B}$, (flexible blocks), and $\phi_{A2}$ (shorter rigid blocks) in the $A_{10}B_{10}A_{4}$ copolymer along the *z*-axis which is perpendicular to the lamellar domains plotted for two values of the repulsion parameter $a_{AB}=30$ (χ=1.53) and $a_{AB}=45$ (χ=6.12). One can see that an increase in the repulsion parameter $a_{AB}$ mainly affects the component fractions within the lamellar B domains. Longer rigid A blocks are expelled from that region, while shorter rigid A blocks are displaced to the boundary between the lamellar A and B domains. We also evaluated the nematic order parameter of the longer A rigid blocks and found it to be weakly positive everywhere with the average value of about 0.12. This value should not be taken too seriously, however, because the orientational order parameter is strongly affected by the layer undulation and the possible local tilt of the rods [45].

### 4.3. The Effect of Polymer Chain Asymmetry on Lamellar Ordering

We focus here on asymmetric rod-coil-rod triblock copolymers where the two rods have different lengths, while, in principle, one would expect that the properties of such a system reduce to those of rod-coil diblock copolymers in the limit of very small length of one of the rods. We have uncovered, however, that the structure of the lamellar phase at high asymmetry of the rods and relatively low concentration of the coils is significantly different from that at low total concentration of rods. In fact, there exist two qualitatively different types of ordering in the lamellar phase. The disordered phase prevails at intermediate values of the rod fraction ratio $f_{r1}/f_{r2}$ and the system undergoes a transition into the lamellar phase both with the increasing temperature and with the decreasing $f_{r1}/f_{r2}$. At relatively high ratio $f_{r1}/f_{r2}>0.5$, the critical value of $f_{r1}/f_{r2}$ is strongly temperature dependent which indicates that the transition into the lamellar phase is promoted by both the phase separation effects, controlled by the Flory-Huggins parameter χ, and by the orientational attraction interaction between rods, which is the primary factor that stabilizes smectic ordering in the theory of low molecular weight liquid crystals.

In contrast, in the case of high rod asymmetry $f_{r1}/f_{r2}<0.4$, the critical value of $f_{r1}/f_{r2}$ is practically temperature independent which means that the lamellar ordering is not affected by the orientational interaction between rods. Moreover, the critical value of $f_{r1}/f_{r2}$ also does not depend on the values of χ, i.e., the transition into the lamellar phase is also not controlled by the repulsion between rods and coils. Then the most likely cause of the translational ordering in this region is the entropy driven spontaneous microphase separation between rods of different lengths.

The peculiar details of unusual structure of lamellar phase, observed at high rods asymmetry, are revealed by the colormaps and profiles of the orientational and translational order parameters presented in Figure 1 and Figure 2. In the region $f_{r1}/f_{r2}>0.5$, all order parameters weakly depend on the asymmetry of the rods and are close to unity, i.e., the lamellar phase is nearly perfectly ordered. In contrast, at higher values of rods asymmetry, all order parameters of short rods dramatically decrease and even turn negative at higher temperatures. Thus the short rods become partially disordered and phase separate away from the long ones. One notes that in contrast to the system simulated in Ref. [56], the segments of the rods are completely equivalent in the present model and hence the phase separation should be driven by some entropy effects. One may assume that in the lamellar phase existing at lower concentration of rods, both short and long rods are mainly located in the same layer and, therefore, the triblock macromolecules are mainly in the hairpin conformation. In contrast, in the unusual lamellar ordering existing at high rod concentration, the short and long rods are located in different layers. Thus the triblock macromolecules are mainly in the extended conformation and overlap three consecutive layers which may result in an enhancement of the mechanical rigidity of the lamellar phase.

These somewhat unexpected results of the density functional theory are independently confirmed by the DPD computer simulations. The DPD simulations have been performed in the region of high rod concentration for different values of the triblock asymmetry and the Flory-Huggins parameter χ. It has been found in this region that both in the case of relatively weak and strong repulsion between rod and coil segments, the shorter rods indeed phase separate from the longer ones despite the fact that they are chemically equivalent. In the case of relatively small χ, the shorter rods mainly reside in the coil block, while in the case of strong segregation, they either form micelles in the coil block or are mainly located at the boundary between the coil block and the block formed by longer rods. In all cases, the density distribution of the shorter rods is rather diffuse which corresponds to the low values of the corresponding translational order parameter. One notes also that the increase of the short rod length may lead to undulation of layers or to the periodic increase of their thickness due to micellae formation. Thus rod-coil-rod triblock copolymers can in principle exhibit a plethora of various interesting structures.

## 5. Conclusions

In this paper, we have developed a molecular-statistical theory of orientationally ordered rod-coil-rod triblock copolymers using the density-functional approach which has been applied by the authors before to the theory of rod-coil diblock copolymers [43]. In this theory, the free energy of the lamellar phase is expressed in terms of the direct correlation functions between segments of different types (i.e., rods and coils) in the reference disordered phase taking into account also the repulsion between rod and coil segments and the orientational interaction between rod segments. The direct correlation functions have been expanded both in Fourier series and in Legendre polynomials keeping the leading terms, and the orientational and translational order parameters of the rod and coil segments, as well as the period of the lamellar phase, have been calculated numerically by direct minimization of the approximate free energy. The incompressibility of the polymer has been accounted for using the Lagrange multiplier technique [44].

It should be noted that the direct correlation functions of block copolymers cannot be calculated in the straightforward way and one has to use Ornstein-Zernike equations which establish a relation between the direct and the total pair correlation functions of a complex fluid. The total correlation functions can then be expressed in terms of density-density correlation functions for a single copolymer chain which can in principle be calculated using the classical polymer theory for a chain composed of rigid rod fragments and Gaussian chains. The Ornstein-Zernike equations are well known in the case of one component simple fluids, but the corresponding equations have never been derived for triblock copolymers which contain segments of three different types possessing different degrees of freedom. In particular, coil segments possess only translational degrees of freedom while rod segments possess both translational and orientational ones. As a result, the rod-coil-rod triblock copolymers should be described by a whole set of such equations. In this paper, we have used the density functional theory to derive nine independent simultaneous Ornstein-Zernike equations which have a nontrivial mathematical structure. These equations cannot be solved analytically, and thus we have used the method of expansion to derive a system of simultaneous linear equations for the expansion coefficients of all direct correlation functions which enter the expression for the free energy of the lamellar phase. This system of equations has been solved numerically and the results have been substituted into the free energy.

Finally, we have presented detailed derivations of the density-density correlation functions for the rod-coil-rod triblock polymer chain. The rod-coil and rod-rod correlation functions have been used before in the theory of rod-coil diblock copolymers [43,44,46] while the correlation function between segments of different rods separated by a coil has been evaluated here for the first time.

One notes that our theory can be further generalized to describe possible structure of the unusual lamellar ordering in greater detail. The presented results are based on the assumption that the waves of densities of different molecular segments are either in phase or in antiphase throughout the phase diagram. In general, one can employ two independent Lagrange multipliers in the numerical minimization of the free energy to account for the possibility of variable phase shifts between them. This is computationally more challenging and will be done in our future publications.

## Data Availability

The data that support the findings of this study are available from the corresponding author upon reasonable request.

## Appendix A. Expansion of the Mean-Field Potentials

In the lamellar phase, all one-particle densities are periodic functions with the period of the phase and hence all other ensemble averages including the effective mean-field potentials are also periodic. Thus one may expand the mean-field potentials in Fourier series taking into account the first harmonics. One notes that it is sufficient to consider only the cosine terms of the corresponding Fourier expansions by appropriately choosing the origin of the coordinate system.

In particular, in this approximation, the contribution from the Flory-Huggins repulsion of rods and coils can be written as

(A1) $$\int \chi \left(r_{12}\right)\delta \rho_{c}\left(r_{2}\right)dr_{2}\approx \rho_{0}f_{c}\psi_{c}\chi \left(q\right)\cos\left(q\cdot r_{1}\right),$$

where the coefficient is defined as

(A2) χ(q)=2∫χ(r)cos(q·r)dr,

and $\psi_{c}$ is the positional order parameter characterizing the translational ordering of the coil fragments (23), which is described by the single-particle distribution function $f_{1}^{\left(c\right)}$ related to the density as $\rho_{c}\left(r\right)=N_{c}f_{1}^{\left(c\right)}\left(r\right)=V\rho_{0}f_{c}f_{1}^{\left(c\right)}\left(r\right)$, where $N_{c}$ is the total number of coil fragments, and $f_{c}$ is the relative fraction of coil fragments.

Similarly,

(A3) $$\int \chi \left(r_{12}\right)\delta \rho_{ri}\left(r_{2},a_{2}\right)dr_{2}da_{2}\approx \rho_{0}f_{ri}\psi_{ri}\chi \left(q\right)\cos\left(q\cdot r_{1}\right),$$

where the positional order parameter of the fragments of rods is given by Equation (24).

In the same approximation, the contribution of the Maier-Saupe-like interactions can approximately be expressed as

(A4) $$\int J\left(r_{12}\right)P_{2}\left(a\cdot a_{2}\right)\delta \rho_{ri}\left(r_{2},a_{2}\right)dr_{2}da_{2}\approx \rho_{0}f_{ri}P_{2}\left(a\cdot k\right)\left[J_{0}S_{i}\right.\left.+J_{2}\sigma_{i}\cos\left(q\cdot r_{1}\right)\right],$$

where

(A5) $$J_{0}=\int J\left(r\right)dr,\;J_{2}=2\int J\left(r\right)\cos\left(q\cdot r\right)dr,$$

and where the nematic order parameter of the rods of type *i* is conventionally introduced by Equation (21) along with the order parameter $\sigma_{i}$ which, according to its definition (22), characterizes the degree of simultaneous orientational and positional ordering of the rods of type *i*.

The arising non-constant contributions to the integral involving the coil-coil correlation function is then expressed as:

(A6) $$\int C_{cc}^{\left(I\right)}\left(r_{12}\right)\delta \rho_{c}\left(r_{2}\right)dr_{2}\approx \rho_{0}f_{c}\psi_{c}C_{cc}^{\left(0\right)}\left(q\right)\cos\left(q\cdot r_{1}\right),$$

where the Fourier coefficient is determined by the integral:

(A7) $$C_{cc}^{\left(0\right)}\left(q\right)=2\int C_{cc}^{\left(I\right)}\left(r\right)\cos\left(q\cdot r\right)dr.$$

Similarly, we expand in cosine series the terms involving the rod-coil correlation function:

(A8) $$\int C_{ric}^{\left(I\right)}\left(r_{12},a_{1}\right)\delta \rho_{c}\left(r_{2}\right)dr_{2}\approx \rho_{0}f_{c}\psi_{c}C_{ric}^{\left(I\right)}\left(q,a_{1}\right)\cos\left(q\cdot r_{1}\right),$$

(A9) $$\int C_{ric}^{\left(I\right)}\left(r_{12},a_{2}\right)\delta \rho_{ri}\left(r_{2},a_{2}\right)dr_{2}da_{2}\approx \rho_{0}f_{ri}{\langle C_{ric}^{\left(I\right)}\left(q,a_{2}\right)\cos\left(q\cdot r_{2}\right)\rangle }_{ri}\;\cos\left(q\cdot r_{1}\right),$$

where the coefficient is introduced as an integral

(A10) $$C_{ric}^{\left(I\right)}\left(q,a\right)=2\int C_{ric}^{\left(I\right)}\left(r,a\right)\cos\left(q\cdot r\right)dr$$

depending on the rod long axis orientation a.

As the latter is a scalar function of the vectors q and a, being defined in the isotropic disordered polymer phase, it depends only on the scalar product (a·q) and, introducing the unit vector k=q/q, we can expand this function in orthogonal Legendre polynomials keeping the first main terms:

(A11) $$C_{ric}^{\left(I\right)}\left(q,a\right)\approx C_{ric}^{\left(0\right)}\left(q\right)+C_{ric}^{\left(2\right)}\left(q\right)P_{2}\left(a\cdot k\right).$$

where the expansion coefficients are expressed as

(A12) $$C_{ric}^{\left(0\right)}\left(q\right)=\frac{1}{2\pi }\int C_{ric}^{\left(I\right)}\left(r,a\right)\cos\left(q\cdot r\right)drda,$$

(A13) $$C_{ric}^{\left(2\right)}\left(q\right)=\frac{5}{2\pi }\int C_{ric}^{\left(I\right)}\left(r,a\right)P_{2}\left(a\cdot k\right)\cos\left(q\cdot r\right)drda.$$

Accordingly, the integrals involving rod-coil correlations reduce to

(A14) $$\int C_{ric}^{\left(I\right)}\left(r_{12},a_{1}\right)\delta \rho_{c}\left(r_{2}\right)dr_{2}\approx \rho_{0}f_{c}\psi_{c}\left[C_{ric}^{\left(0\right)}\left(q\right)\cos\left(q\cdot r_{1}\right)+C_{ric}^{\left(2\right)}\left(q\right)P_{2}\left(a_{1}\cdot k\right)\cos\left(q\cdot r_{1}\right)\right],$$

(A15) $$\int C_{ric}^{\left(I\right)}\left(r_{12},a_{2}\right)\delta \rho_{ri}\left(r_{2},a_{2}\right)dr_{2}da_{2}\approx \rho_{0}f_{ri}\left[\psi_{ri}C_{ric}^{\left(0\right)}\left(q\right)+\sigma_{i}C_{ric}^{\left(2\right)}\left(q\right)\right]\;\cos\left(q\cdot r_{1}\right).$$

The terms involving the integrals of the correlations of rod fragments of different types can be expanded in Fourier series as:

(A16) $$\begin{array}{c} \int C_{r1r2}^{\left(I\right)}\left(r_{12},a_{1},a_{2}\right)\delta \rho_{ri}\left(r_{2},a_{2}\right)dr_{2}da_{2}\\ \;\approx \rho_{0}f_{ri}{\langle {\bar{C}}_{r1r2}\left(a_{1},a_{2}\right)\rangle }_{ri}+\rho_{0}f_{ri}{\langle C_{r1r2}^{\left(I\right)}\left(q,a_{1},a_{2}\right)\cos\left(q\cdot r_{2}\right)\rangle }_{ri}\cos\left(q\cdot r_{1}\right) \end{array}$$

where again the averaging ${\langle \cdots \rangle }_{ri}$ is performed with the single-particle distribution function $f_{1}^{\left(ri\right)}\left(r_{2},a_{2}\right)$ and involves the functions:

(A17) $$C_{r1r2}^{\left(I\right)}\left(q,a_{1},a_{2}\right)=2\int C_{r1r2}^{\left(I\right)}\left(r,a_{1},a_{2}\right)\cos\left(q\cdot r\right)dr,$$

(A18) $${\bar{C}}_{r1r2}\left(a_{1},a_{2}\right)=\int C_{r1r2}^{\left(I\right)}\left(r,a_{1},a_{2}\right)dr.$$

Expanding the latter two integrals in Legendre polynomials enables one to express them as

(A19) $$C_{r1r2}^{\left(I\right)}\left(q,a_{1},a_{2}\right)\approx C_{r1r2}^{\left(0\right)}\left(q\right)+C_{r1r2}^{\left(2\right)}\left(q\right)\left[P_{2}\left(a_{1}\cdot k\right)+P_{2}\left(a_{2}\cdot k\right)\right]+C_{r1r2}^{\left(3\right)}\left(q\right)P_{2}\left(a_{1}\cdot a_{2}\right),$$

(A20) $${\bar{C}}_{r1r2}\left(a_{1},a_{2}\right)=C_{r1r2}^{\left(4\right)}P_{2}\left(a_{1}\cdot a_{2}\right),$$

where the coefficients are defined as the following integrals:

(A21) $$C_{r1r2}^{\left(0\right)}\left(q\right)=\frac{1}{8\pi^{2}}\int C_{r1r2}^{\left(I\right)}\left(r,a_{1},a_{2}\right)\cos\left(q\cdot r\right)drda_{1}da_{2},$$

(A22) $$C_{r1r2}^{\left(2\right)}\left(q\right)=\frac{5}{8\pi^{2}}\int C_{r1r2}^{\left(I\right)}\left(r,a_{1},a_{2}\right)P_{2}\left(a_{1}\cdot k\right)\cos\left(q\cdot r\right)drda_{1}da_{2},$$

(A23) $$C_{r1r2}^{\left(3\right)}\left(q\right)=\frac{5}{8\pi^{2}}\int C_{r1r2}^{\left(I\right)}\left(r,a_{1},a_{2}\right)P_{2}\left(a_{1}\cdot a_{2}\right)\cos\left(q\cdot r\right)drda_{1}da_{2},$$

(A24) $$C_{r1r2}^{\left(4\right)}=\frac{5}{16\pi^{2}}\int C_{r1r2}^{\left(I\right)}\left(r,a_{1},a_{2}\right)P_{2}\left(a_{1}\cdot a_{2}\right)drda_{1}da_{2}.$$

This reduces the term (A16) to:

(A25) $$\begin{array}{c} \int C_{r1r2}^{\left(I\right)}\left(r_{12},a_{1},a_{2}\right)\delta \rho_{ri}\left(r_{2},a_{2}\right)dr_{2}da_{2}\\ \;\approx \rho_{0}f_{ri}S_{i}P_{2}\left(a_{1}\cdot k\right)C_{r1r2}^{\left(4\right)}+\rho_{0}f_{ri}\psi_{i}C_{r1r2}^{\left(0\right)}\left(q\right)\cos\left(q\cdot r_{1}\right)+\rho_{0}f_{ri}\psi_{ri}C_{r1r2}^{\left(2\right)}\left(q\right)P_{2}\left(a_{1}\cdot k\right)\cos\left(q\cdot r_{1}\right)\\ \;+\rho_{0}f_{ri}\sigma_{i}C_{r1r2}^{\left(2\right)}\left(q\right)\cos\left(q\cdot r_{1}\right)+\rho_{0}f_{ri}\sigma_{i}C_{r1r2}^{\left(3\right)}\left(q\right)P_{2}\left(a_{1}\cdot k\right)\cos\left(q\cdot r_{1}\right). \end{array}$$

Finally, the main term of the Fourier expansion of the contribution of the correlations functions between rods of the same type is expressed as:

(A26) $$\int C_{riri}^{\left(I\right)}\left(r_{12},a_{1}\right)\delta \rho_{ri}\left(r_{2},a_{1}\right)dr_{2}\approx \rho_{0}f_{ri}\cos\left(q\cdot r_{1}\right)C_{riri}^{\left(I\right)}\left(q,a_{1}\right)\int f_{1}^{\left(ri\right)}\left(r_{2},a_{1}\right)\cos\left(q\cdot r_{2}\right)dr_{2},$$

where the Fourier component of the correlation function is introduced similarly to those above:

(A27) $$C_{riri}^{\left(I\right)}\left(q,a_{1}\right)=2\int C_{riri}^{\left(I\right)}\left(r,a_{1}\right)\cos\left(q\cdot r\right)dr.$$

The main terms of the Legendre polynomial expansion of the latter are

(A28) $$C_{riri}^{\left(I\right)}\left(q,a_{1}\right)\approx 4\pi C_{riri}^{\left(0\right)}\left(q\right)+4\pi C_{riri}^{\left(2\right)}\left(q\right)P_{2}\left(a_{1}\cdot k\right).$$

with the coefficients defined as:

(A29) $$C_{riri}^{\left(0\right)}\left(q\right)=\frac{1}{8\pi^{2}}\int C_{riri}^{\left(I\right)}\left(r,a\right)\cos\left(q\cdot r\right)drda,$$

(A30) $$C_{riri}^{\left(2\right)}\left(q\right)=\frac{5}{8\pi^{2}}\int C_{riri}^{\left(I\right)}\left(r,a\right)P_{2}\left(a\cdot k\right)\cos\left(q\cdot r\right)drda.$$

Similar Legendre polynomial expansion of the Fourier coefficient in Equation (A26) reads as:

(A31) $$\begin{array}{c} C_{riri}^{\left(I\right)}\left(q,a_{1}\right)\int f_{1}^{\left(ri\right)}\left(r_{2},a_{1}\right)\cos\left(q\cdot r_{2}\right)dr_{2}\\ \;\approx \frac{1}{4\pi }\int C_{riri}^{\left(I\right)}\left(q,a_{2}\right)f_{1}^{\left(ri\right)}\left(r_{2},a_{2}\right)\cos\left(q\cdot r_{2}\right)dr_{2}da_{2}\\ \;+\frac{5}{4\pi }P_{2}\left(a_{1}\cdot k\right)\int C_{riri}^{\left(I\right)}\left(q,a_{2}\right)f_{1}^{\left(ri\right)}\left(r_{2},a_{2}\right)\cos\left(q\cdot r_{2}\right)P_{2}\left(a_{2}\cdot k\right)dr_{2}da_{2}. \end{array}$$

Substituting here Equation (A28) yields:

(A32) $$\begin{array}{c} C_{riri}^{\left(I\right)}\left(q,a_{1}\right)\int f_{1}^{\left(ri\right)}\left(r_{2},a_{1}\right)\cos\left(q\cdot r_{2}\right)dr_{2}\\ \;\approx C_{riri}^{\left(0\right)}\left(q\right)\psi_{ri}+C_{riri}^{\left(2\right)}\left(q\right)\sigma_{i}+5P_{2}\left(a_{1}\cdot k\right)C_{riri}^{\left(0\right)}\left(q\right)\sigma_{i}\\ \;+5P_{2}\left(a_{1}\cdot k\right)C_{riri}^{\left(2\right)}\left(q\right){\langle P_{2}^{2}\left(a_{1}\cdot k\right)\cos\left(q\cdot r_{2}\right)\rangle }_{ri}. \end{array}$$

The last term here is expressed as an expansion in Legendre polynomials (the first constant term here is essential for the free energy self consistence):

(A33) $$\begin{array}{c} P_{2}^{2}\left(a_{1}\cdot k\right)=\frac{1}{4\pi }\int P_{2}^{2}\left(a_{2}\cdot k\right)da_{2}+\frac{5}{4\pi }P_{2}\left(a_{1}\cdot k\right)\int P_{2}^{2}\left(a_{2}\cdot k\right)P_{2}\left(a_{2}\cdot k\right)da_{2}+\ldots \\ =\frac{1}{5}+\frac{2}{7}P_{2}\left(a_{1}\cdot k\right)+\ldots \end{array}$$

Therefore, neglecting higher order Legendre polynomials one can express the contribution (A26) to the mean-field potentials in Equations (12) and (13) as:

(A34) $$\begin{array}{c} \int C_{riri}^{\left(I\right)}\left(r_{12},a_{1}\right)\delta \rho_{ri}\left(r_{2},a_{1}\right)dr_{2}\approx \rho_{0}f_{ri}C_{riri}^{\left(0\right)}\left(q\right)\psi_{ri}\cos\left(q\cdot r_{1}\right)\\ \;+\rho_{0}f_{ri}C_{riri}^{\left(2\right)}\left(q\right)\sigma_{i}\cos\left(q\cdot r_{1}\right)+\rho_{0}f_{ri}C_{riri}^{\left(2\right)}\left(q\right)\psi_{ri}P_{2}\left(a_{1}\cdot k\right)\cos\left(q\cdot r_{1}\right)\\ \;+5\rho_{0}f_{ri}\left[C_{riri}^{\left(0\right)}\left(q\right)+\frac{2}{7}C_{riri}^{\left(2\right)}\left(q\right)\right]\sigma_{i}P_{2}\left(a_{1}\cdot k\right)\cos\left(q\cdot r_{1}\right). \end{array}$$

Substituting the expansions (A6), (A14), (A15), (A25) and (A34) into the densities (11)–(13) transforms the free energy of the lamellar phase of rod-coil-rod triblock copolymers (6) into a self-consistent form of Equation (17).

## Appendix B. Derivation of the Ornstein-Zernike Equations for Rod-Coil-Rod Triblock Copolymers

Here we start with the generalized Legendre transformation equation for the grand canonical potential $\Omega [U_{r1}\left(x\right),U_{r2}\left(x\right),U_{c}\left(r\right)]$ which depends on the external field acting on chain segments of different types, and on the corresponding chemical potentials $\mu_{\nu }^{0},\left(\nu =r1,r2,c\right)$, and which is similar to (1):

(A35) $$\beta F\left[\rho_{r1}\left(x\right),\rho_{r2}\left(x\right),\rho_{c}\left(r\right)\right]=\sum_{\nu }\int \rho_{\nu }\left(x\right)\phi_{\nu }\left(x\right)dx-\Omega ,$$

where

(A36) $$\phi_{\nu }=\beta \mu_{\nu }-\beta U_{\nu }^{e},$$

and where *F* is the free energy functional which depends on the number densities of the segments of the two rods and the coil. The functional derivative of *F* with respect to $\rho_{\nu }$ is [66]:

(A37) $$\beta \frac{\delta F}{\delta \rho_{\nu }\left(x_{\nu }\right)}=\phi_{\nu }\left(x_{\nu }\right),$$

It follows from the definition of the grand canonical potential (see, for example, Ref. [38]) that

(A38) $$\beta \frac{\delta \Omega }{\delta \phi_{\nu }\left(x_{\nu }\right)}=\rho_{\nu }\left(x_{\nu }\right),$$

and

(A39) $$\begin{array}{c} \frac{\delta \rho_{\nu }}{\delta \phi_{\mu }\left(x_{\mu }\right)}=\beta \frac{\delta^{2}\Omega }{\delta \phi_{\nu }\left(x_{\nu }\right)\delta \phi_{\mu }\left(x_{\mu }\right)}=\langle \delta \rho_{\nu }\left(x_{\nu }\right)\delta \rho_{\mu }\left(x_{\mu }\right)\rangle \\ \;=\delta_{\mu \nu }\rho_{\mu }\left(x_{\mu }\right)\delta \left(x_{\mu }-x_{\nu }\right)+\rho_{\nu }\left(x_{\nu }\right)\rho_{\mu }\left(x_{\mu }\right)h_{\nu \mu }\left(x_{\nu },x_{\mu }\right), \end{array}$$

where $h_{\nu \mu }\left(x_{\nu },x_{\mu }\right)$ are the corresponding total pair correlation functions.

On the other hand, the free energy *F* can be written as a sum of two terms as in Equation (3):

(A40) F=W+H,

where *W* is the free energy of the system without intermolecular interactions:

(A41) $$\beta W=\sum_{\nu }\int \rho_{\nu }\left(x_{1,\nu }\right)\left(\ln\rho_{\nu }\left(x_{1,\nu }\right)\Lambda +\beta U_{\nu }^{e}-1\right)\;dx_{1,\nu },$$

and where the second functional derivatives of *H* with respect to $\rho_{\nu }$ are related to the direct correlation functions $C_{\mu \nu }\left(x_{\mu },x_{\nu }\right)$ by Equation (5).

From (A37), (A41) and (5) one obtains:

(A42) $$\frac{\delta \phi_{\nu }\left(x_{1,\nu }\right)}{\delta \rho_{\mu }\left(x_{2,\mu }\right)}=\rho {\left(x_{1,\nu }\right)}^{-1}\delta_{\nu ,\mu }\delta \left(x_{1,\nu }-x_{2,\mu }\right)-C_{\mu \nu }\left(x_{1,\nu },x_{2,\mu }\right).$$

One notes that the functions $\frac{\delta \phi_{\nu }\left(x_{1,\nu }\right)}{\delta \rho_{\mu }\left(x_{2,\mu }\right)}$ and $\frac{\delta \rho_{\nu }}{\delta \phi_{\mu }\left(x_{\mu }\right)}$ given by (A42) and (A39), respectively, are the inverse derivatives of each other and in the discrete space can be considered as the inverse matrices. Hence the product of these matrices should be equal to the generalized unit matrix:

(A43) $$\begin{array}{c} \sum_{\gamma }\int d^{3}x_{3}\left(\rho {\left(x_{1,\nu }\right)}^{-1}\delta_{\nu ,\gamma }\delta \left(x_{1,\nu }-x_{3,\gamma }\right)-C_{\gamma \nu }\left(x_{1,\nu },x_{3,\gamma }\right)\right)\\ \;\times \delta_{\mu \gamma }\rho_{\mu }\left(x_{\mu }\right)\delta \left(x_{\mu }-x_{\gamma }\right)+\rho_{\gamma }\left(x_{\gamma }\right)\rho_{\mu }\left(x_{\mu }\right)h_{\gamma \mu }\left(x_{\gamma },x_{\mu }\right)=\delta_{\nu ,\mu }\delta \left(x_{1,\nu }-x_{2,\mu }\right). \end{array}$$

From (A43) one readily obtains the general Ornstein-Zernike equations for the rod-coil-rod triblock copolymers:

(A44) $$h_{\mu \nu }\left(x_{1,\mu },x_{2,\nu }\right)=C_{\mu \nu }\left(x_{1,\mu },x_{2,\nu }\right)+\sum_{\gamma }\int d^{3}x_{3}C_{\mu \gamma }\left(x_{1,\mu },x_{3,\gamma }\right)h_{\gamma \nu }\left(x_{3,\gamma },x_{2,\nu }\right)\rho_{\gamma }\left(x_{3}\right),$$

where x=(r,a) for rod segments and x=r for coil ones.

As the both definitions of the direct and total correlation functions, (5) and (A39) correspondingly, are symmetric with respect to the index permutations, so are the correlation functions: $C_{\mu \nu }\left(x_{\mu },x_{\nu }\right)=C_{\nu \mu }\left(x_{\nu },x_{\mu }\right)$ and $h_{\mu \nu }\left(x_{\mu },x_{\nu }\right)=h_{\nu \mu }\left(x_{\nu },x_{\mu }\right)$. Therefore, another form of the Ornstein-Zernike equation:

(A45) $$h_{\mu \nu }\left(x_{1,\mu },x_{2,\nu }\right)=C_{\mu \nu }\left(x_{1,\mu },x_{2,\nu }\right)+\sum_{\gamma }\int d^{3}x_{3}h_{\mu \gamma }\left(x_{1,\mu },x_{3,\gamma }\right)C_{\gamma \nu }\left(x_{3,\gamma },x_{2,\nu }\right)\rho_{\gamma }\left(x_{3}\right),$$

is of equally validity.

The triblock polymer free energy expressed by Equation (17) is determined by the direct correlation functions $C^{\left(I\right)}$ between different fragments of a single polymer chain in the disordered phase. The homogeneity of the disordered phase implies that all the densities are constant:

(A46) $$\rho_{cc}=\rho_{0}f_{c},\;\rho_{ri}=\frac{1}{4\pi }\rho_{0}f_{ri}.$$

Therefore, the direct and total correlations of the coil monomers depend only on the distance between them and obey the corresponding Ornstein-Zernike equation Equation (27). Analogously, for the Ornstein-Zernike equations describing the correlation of coil monomers with those of the rods 1, the general (A45) yields Equation (28). Taking into account that all segments of the same rod of type *i* are identically oriented along a unit vector $a_{i}$, the Ornstein-Zernike equations for them can be written as Equation (29). Finally, the remaining Ornstein-Zernike equation for the correlations of monomers of different rods can be obtained from Equation (A45) as given by Equation (30).

Taking into account that in the isotropic phase $\rho_{\gamma }\left(r,a\right)=f_{\gamma }\rho_{0}/4\pi$ one obtains in the Fourier representation:

(A47) $$h_{\mu \nu }\left(q,a_{1},a_{2}\right)=C_{\mu \nu }\left(q,a_{1},a_{2}\right)+\frac{1}{4\pi }\rho_{0}\sum_{\gamma }f_{\gamma }\int d^{2}a_{3}C_{\mu \gamma }\left(q,a_{1},a_{3}\right)h_{\gamma \nu }\left(q,a_{3},a_{2}\right)$$

where in the isotropic phase the function $h_{\mu ,\nu }$ is defined by the equation:

(A48) $$\langle \delta \rho_{\nu }\left(q,a_{1}\right)\delta \rho_{\mu }\left(q,a_{2}\right)\rangle =\delta_{\mu \nu }f_{\mu }\frac{1}{4\pi }\delta \left(r_{2}-r_{1}\right)\delta \left(a_{1}-a_{2}\right)+\frac{1}{{\left(4\pi \right)}^{2}}f_{\mu }f_{\nu }h_{\mu \nu }\left(q,a_{1},a_{2}\right).$$

The main part of the free energy (17) is determined by the Fourier coefficients of the direct correlation functions all defined in a similar way as

(A49) $$C_{\mu \nu }^{\left(I\right)}\left(q,\ldots \right)=2\int C_{\mu \nu }^{\left(I\right)}\left(r,\ldots \right)\cos\left(q\cdot r\right)dr.$$

The Fourier harmonics of the total correlation functions can be similarly defined as:

(A50) $$h_{\mu \nu }\left(q,\ldots \right)=2\int h_{\mu \nu }\left(r,\ldots \right)\cos\left(q\cdot r\right)dr.$$

Now it is straightforward to derive the Fourier transforms of the Ornstein-Zernike equations in which all convoluted spatial integrals transform to the products of the Fourier coefficients. Thus Equation (27) then transforms to:

(A51) $$\begin{array}{c} h_{cc}\left(q\right)=C_{cc}^{\left(I\right)}\left(q\right)+\frac{1}{8\pi }\rho_{0}f_{r1}\int C_{r1c}^{\left(I\right)}\left(q,a_{1}\right)h_{r1c}\left(q,a_{1}\right)da_{1}\\ \;+\frac{1}{8\pi }\rho_{0}f_{r2}\int C_{r2c}^{\left(I\right)}\left(q,a_{2}\right)h_{r2c}\left(q,a_{2}\right)da_{2}+\frac{1}{2}\rho_{0}f_{c}C_{cc}^{\left(I\right)}\left(q\right)h_{cc}\left(q\right). \end{array}$$

Analogously, Equations (28) and its counterpart for rods 2 can be written as:

(A52) $$\begin{array}{c} h_{r1c}\left(q,a_{1}\right)=C_{r1c}^{\left(I\right)}\left(q,a_{1}\right)+\frac{1}{8\pi }\rho_{0}f_{r1}h_{r1r1}\left(q,a_{1}\right)C_{r1c}^{\left(I\right)}\left(q,a_{1}\right)\\ \;+\frac{1}{8\pi }\rho_{0}f_{r2}\int h_{r1r2}\left(q,a_{1},a_{2}\right)C_{r2c}^{\left(I\right)}\left(q,a_{2}\right)da_{2}+\frac{1}{2}\rho_{0}f_{c}h_{r1c}\left(q,a_{1}\right)C_{cc}^{\left(I\right)}\left(q\right), \end{array}$$

(A53) $$\begin{array}{c} h_{r2c}\left(q,a_{2}\right)=C_{r2c}^{\left(I\right)}\left(q,a_{2}\right)+\frac{1}{8\pi }\rho_{0}f_{r2}h_{r2r2}\left(q,a_{2}\right)C_{r2c}^{\left(I\right)}\left(q,a_{2}\right)\\ \;+\frac{1}{8\pi }\rho_{0}f_{r1}\int h_{r1r2}\left(q,a_{1},a_{2}\right)C_{r1c}^{\left(I\right)}\left(q,a_{1}\right)da_{1}+\frac{1}{2}\rho_{0}f_{c}h_{r2c}\left(q,a_{2}\right)C_{cc}^{\left(I\right)}\left(q\right), \end{array}$$

while Equations (29) yields

(A54) $$\begin{array}{c} h_{r1r1}\left(q,a_{1}\right)=C_{r1r1}^{\left(I\right)}\left(q,a_{1}\right)+\frac{1}{8\pi }\rho_{0}f_{r1}C_{r1r1}^{\left(I\right)}\left(q,a_{1}\right)h_{r1r1}\left(q,a_{1}\right)\\ \;+\frac{1}{8\pi }\rho_{0}f_{r2}\int C_{r1r2}^{\left(I\right)}\left(q,a_{1},a_{2}\right)h_{r1r2}\left(q,a_{1},a_{2}\right)da_{2}+\frac{1}{2}\rho_{0}f_{c}C_{r1c}^{\left(I\right)}\left(q,a_{1}\right)h_{r1c}\left(q,a_{1}\right), \end{array}$$

(A55) $$\begin{array}{c} h_{r2r2}\left(r,a_{2}\right)=C_{r2r2}^{\left(I\right)}\left(r,a_{2},a_{2}^{\prime }\right)+\frac{1}{8\pi }\rho_{0}f_{r2}C_{r2r2}^{\left(I\right)}\left(q,a_{2}\right)h_{r2r2}\left(q,a_{2}\right)\\ \;+\frac{1}{8\pi }\rho_{0}f_{r1}\int C_{r1r2}^{\left(I\right)}\left(q,a_{1},a_{2}\right)h_{r1r2}\left(q,a_{1},a_{2}\right)da_{1}+\frac{1}{2}\rho_{0}f_{c}C_{r2c}^{\left(I\right)}\left(q,a_{2}\right)h_{r2c}\left(q,a_{2}\right). \end{array}$$

Finally, Equation (30) allows obtaining:

(A56) $$\begin{array}{c} h_{r1r2}\left(q,a_{1},a_{2}\right)=C_{r1r2}^{\left(I\right)}\left(q,a_{1},a_{2}\right)+\frac{1}{8\pi }\rho_{0}f_{r1}h_{r1r1}\left(q,a_{1}\right)C_{r1r2}^{\left(I\right)}\left(q,a_{1},a_{2}\right)\\ \;+\frac{1}{8\pi }\rho_{0}f_{r2}h_{r1r2}\left(q,a_{1},a_{2}\right)C_{r2r2}^{\left(I\right)}\left(q,a_{2}\right)+\frac{1}{2}\rho_{0}f_{c}h_{r1c}\left(q,a_{1}\right)C_{r2c}^{\left(I\right)}\left(q,a_{2}\right). \end{array}$$

## Appendix C. Solution of the Ornstein-Zernike Equations

Ornstein-Zernike equations for rod-coil-rod triblock copolymers cannot be solved analytically. On the other hand, the approximate expression for the free energy of the lamellar phase contains only the low order expansion coefficients of the direct correlation functions. It is possible to derive approximate equations for these coefficients substituting Legendre polynomials expansions of the direct correlations into the corresponding Ornstein-Zernike equations. In particular, using the expansion (A11) it is possible to transform the integral Equation (A51) into an algebraic equation:

(A57) $$\begin{array}{c} h_{cc}\left(q\right)=C_{cc}^{\left(0\right)}\left(q\right)\left[1+\frac{1}{2}\rho_{0}f_{c}h_{cc}\left(q\right)\right]+\frac{1}{2}\rho_{0}f_{r1}C_{r1c}^{\left(0\right)}\left(q\right)h_{r1c}^{\left(0\right)}\left(q\right)+\frac{1}{2}\rho_{0}f_{r2}C_{r2c}^{\left(0\right)}\left(q\right)h_{r2c}^{\left(0\right)}\left(q\right)\\ +\frac{1}{2}\rho_{0}f_{r1}C_{r1c}^{\left(2\right)}\left(q\right)h_{r1c}^{\left(2\right)}\left(q\right)+\frac{1}{2}\rho_{0}f_{r2}C_{r2c}^{\left(2\right)}\left(q\right)h_{r2c}^{\left(2\right)}\left(q\right), \end{array}$$

where the coefficients are introduced as

(A58) $$h_{ric}^{\left(0\right)}\left(q\right)=\frac{1}{4\pi }\int h_{ric}\left(q,a\right)da,$$

(A59) $$h_{ric}^{\left(2\right)}\left(q\right)=\frac{1}{4\pi }\int h_{ric}\left(q,a\right)P_{2}\left(a\cdot k\right)da.$$

Next we integrate Equations (A52) and (A53) over the corresponding orientations $a_{i}$, substitute again Equation (A11) and obtain:

(A60) $$\begin{array}{c} h_{r1c}^{\left(0\right)}\left(q\right)=C_{r1c}^{\left(0\right)}\left(q\right)\left[1+\frac{1}{8\pi }\rho_{0}f_{r1}h_{r1r1}^{\left(0\right)}\left(q\right)\right]+\frac{1}{8\pi }\rho_{0}f_{r1}C_{r1c}^{\left(2\right)}\left(q\right)h_{r1r1}^{\left(2\right)}\left(q\right)\\ \;+\frac{1}{2}\rho_{0}f_{r2}C_{r2c}^{\left(0\right)}\left(q\right)h_{r1r2}^{\left(0\right)}\left(q\right)+\frac{1}{2}\rho_{0}f_{r2}C_{r2c}^{\left(2\right)}\left(q\right)h_{r1r2}^{\left(2\right)}\left(q\right)+\frac{1}{2}\rho_{0}f_{c}C_{cc}^{\left(0\right)}\left(q\right)h_{r1c}^{\left(0\right)}\left(q\right), \end{array}$$

(A61) $$\begin{array}{c} h_{r2c}^{\left(0\right)}\left(q\right)=C_{r2c}^{\left(0\right)}\left(q\right)\left[1+\frac{1}{8\pi }\rho_{0}f_{r2}h_{r2r2}^{\left(0\right)}\left(q\right)\right]+\frac{1}{8\pi }\rho_{0}f_{r2}C_{r2c}^{\left(2\right)}\left(q\right)h_{r2r2}^{\left(2\right)}\left(q\right)\\ \;+\frac{1}{2}\rho_{0}f_{r1}C_{r1c}^{\left(0\right)}\left(q\right)h_{r1r2}^{\left(0\right)}\left(q\right)+\frac{1}{2}\rho_{0}f_{r1}C_{r1c}^{\left(2\right)}\left(q\right)h_{r1r2}^{\left(2\right)}\left(q\right)+\frac{1}{2}\rho_{0}f_{c}C_{cc}^{\left(0\right)}\left(q\right)h_{r2c}^{\left(0\right)}\left(q\right). \end{array}$$

The same procedure applied to Equations (A52) and (A53) multiplied by $P_{2}\left(a_{i}\cdot k\right)$ yields

(A62) $$\begin{array}{c} h_{r1c}^{\left(2\right)}\left(q\right)=\frac{1}{5}C_{r1c}^{\left(2\right)}\left(q\right)+\frac{1}{8\pi }\rho_{0}f_{r1}C_{r1c}^{\left(0\right)}\left(q\right)h_{r1r1}^{\left(2\right)}\left(q\right)+\frac{1}{8\pi }\rho_{0}f_{r1}C_{r1c}^{\left(2\right)}\left(q\right)h_{r1r1}^{\left(5\right)}\left(q\right)\\ \;+\frac{1}{2}\rho_{0}f_{r2}C_{r2c}^{\left(0\right)}\left(q\right)h_{r1r2}^{\left(2\right)}\left(q\right)+\frac{1}{2}\rho_{0}f_{r2}C_{r2c}^{\left(2\right)}\left(q\right)h_{r1r2}^{\left(5\right)}\left(q\right)+\frac{1}{2}\rho_{0}f_{c}C_{cc}^{\left(0\right)}\left(q\right)h_{r1c}^{\left(2\right)}\left(q\right), \end{array}$$

(A63) $$\begin{array}{c} h_{r2c}^{\left(2\right)}\left(q\right)=\frac{1}{5}C_{r2c}^{\left(2\right)}\left(q\right)+\frac{1}{8\pi }\rho_{0}f_{r2}C_{r2c}^{\left(0\right)}\left(q\right)h_{r2r2}^{\left(2\right)}\left(q\right)+\frac{1}{8\pi }\rho_{0}f_{r2}C_{r2c}^{\left(2\right)}\left(q\right)h_{r2r2}^{\left(5\right)}\left(q\right)\\ \;+\frac{1}{2}\rho_{0}f_{r1}C_{r1c}^{\left(0\right)}\left(q\right)h_{r1r2}^{\left(2\right)}\left(q\right)+\frac{1}{2}\rho_{0}f_{r1}C_{r1c}^{\left(2\right)}\left(q\right)h_{r1r2}^{\left(5\right)}\left(q\right)+\frac{1}{2}\rho_{0}f_{c}C_{cc}^{\left(0\right)}\left(q\right)h_{r2c}^{\left(2\right)}\left(q\right), \end{array}$$

where the coefficients are defined as:

(A64) $$h_{riri}^{\left(0\right)}\left(q\right)=\frac{1}{4\pi }\int h_{riri}\left(q,a\right)da,$$

(A65) $$h_{riri}^{\left(2\right)}\left(q\right)=\frac{1}{4\pi }\int h_{riri}\left(q,a\right)P_{2}\left(a\cdot k\right)da,$$

(A66) $$h_{riri}^{\left(5\right)}\left(q\right)=\frac{1}{4\pi }\int h_{riri}\left(q,a\right)P_{2}^{2}\left(a\cdot k\right)da,$$

(A67) $$h_{r1r2}^{\left(0\right)}\left(q\right)=\frac{1}{16\pi^{2}}\int h_{r1r2}\left(q,a_{1},a_{2}\right)da_{1}da_{2},$$

(A68) $$h_{r1r2}^{\left(2\right)}\left(q\right)=\frac{1}{16\pi^{2}}\int h_{r1r2}\left(q,a_{1},a_{2}\right)P_{2}\left(a_{1}\cdot k\right)da_{1}da_{2},$$

(A69) $$h_{r1r2}^{\left(5\right)}\left(q\right)=\frac{1}{16\pi^{2}}\int h_{r1r2}\left(q,a_{1},a_{2}\right)P_{2}\left(a_{1}\cdot k\right)P_{2}\left(a_{2}\cdot k\right)da_{1}da_{2}.$$

Altogether, Equations (A57) and (A60)–(A63) form an enclosed system that can be solved to obtain independently 5 parameters $C_{cc}^{\left(0\right)}\left(q\right)$, $C_{r1c}^{\left(0\right)}\left(q\right)$, $C_{r2c}^{\left(0\right)}\left(q\right)$, $C_{r1c}^{\left(2\right)}\left(q\right)$, and $C_{r2c}^{\left(2\right)}\left(q\right)$.

Now, substituting the expansion of the direct correlation functions given by Equations (A11), (A19) and (A28) into Equation (A56), and integrating over $a_{1}$ and $a_{2}$, one obtains:

(A70) $$\begin{array}{c} h_{r1r2}^{\left(0\right)}\left(q\right)=C_{r1r2}^{\left(0\right)}\left(q\right)+\frac{1}{8\pi }\rho_{0}f_{r1}C_{r1r2}^{\left(0\right)}\left(q\right)h_{r1r1}^{\left(0\right)}\left(q\right)+\frac{1}{8\pi }\rho_{0}f_{r1}C_{r1r2}^{\left(2\right)}\left(q\right)h_{r1r1}^{\left(2\right)}\left(q\right)\\ \;+\frac{1}{2}\rho_{0}f_{r2}C_{r2r2}^{\left(0\right)}\left(q\right)h_{r1r2}^{\left(0\right)}\left(q\right)+\frac{1}{2}\rho_{0}f_{r2}C_{r2r2}^{\left(2\right)}\left(q\right)h_{r1r2}^{\left(2\right)}\left(q\right)+\frac{1}{2}\rho_{0}f_{c}h_{r1c}^{\left(0\right)}\left(q\right)C_{r2c}^{\left(0\right)}\left(q\right). \end{array}$$

Similarly, we integrate Equation (A56) multiplied by $P_{2}\left(a_{1}\cdot k\right)$ and obtain:

(A71) $$\begin{array}{c} h_{r1r2}^{\left(2\right)}\left(q\right)=\frac{1}{5}C_{r1r2}^{\left(2\right)}\left(q\right)+\frac{1}{8\pi }\rho_{0}f_{r1}h_{r1r1}^{\left(2\right)}\left(q\right)C_{r1r2}^{\left(0\right)}\left(q\right)+\frac{1}{8\pi }\rho_{0}f_{r1}h_{r1r1}^{\left(5\right)}\left(q\right)C_{r1r2}^{\left(2\right)}\left(q\right)\\ \;+\frac{1}{2}\rho_{0}f_{r2}h_{r1r2}^{\left(2\right)}\left(q\right)C_{r2r2}^{\left(0\right)}\left(q\right)+\frac{1}{2}\rho_{0}f_{r2}h_{r1r2}^{\left(5\right)}\left(q\right)C_{r2r2}^{\left(2\right)}\left(q\right)+\frac{1}{2}\rho_{0}f_{c}h_{r1c}^{\left(2\right)}\left(q\right)C_{r2c}^{\left(0\right)}\left(q\right). \end{array}$$

Integrating Equation (A56) multiplied by $P_{2}\left(a_{1}\cdot a_{2}\right)$ one also obtains

(A72) $$\begin{array}{c} h_{r1r2}^{\left(3\right)}\left(q\right)=\frac{1}{5}C_{r1r2}^{\left(3\right)}\left(q\right)+\frac{1}{40\pi }\rho_{0}f_{r1}h_{r1r1}^{\left(2\right)}\left(q\right)C_{r1r2}^{\left(2\right)}\left(q\right)+\frac{1}{40\pi }\rho_{0}f_{r1}h_{r1r1}^{\left(0\right)}\left(q\right)C_{r1r2}^{\left(3\right)}\left(q\right)\\ \;+\frac{1}{2}\rho_{0}f_{r2}h_{r1r2}^{\left(3\right)}\left(q\right)C_{r2r2}^{\left(0\right)}\left(q\right)+\frac{1}{2}\rho_{0}f_{r2}h_{r1r2}^{\left(6\right)}\left(q\right)C_{r2r2}^{\left(2\right)}\left(q\right)+\frac{1}{10}\rho_{0}f_{c}h_{r1c}^{\left(2\right)}\left(q\right)C_{r2c}^{\left(2\right)}\left(q\right), \end{array}$$

where we have used the relation $\int P_{2}\left(u\cdot a\right)P_{2}\left(v\cdot a\right)da=\frac{4\pi }{5}P_{2}\left(v\cdot u\right)$ valid for arbitrary unit vectors u and v, and another coefficient is introduced as

(A73) $$h_{r1r2}^{\left(6\right)}\left(q\right)=\frac{1}{16\pi^{2}}\int h_{r1r2}\left(q,a_{1},a_{2}\right)P_{2}\left(a_{1}\cdot k\right)P_{2}\left(a_{1}\cdot a_{2}\right)da_{1}da_{2}.$$

In a similar manner, integrating Equations (A54) and (A55) over all orientations of the unit vector $a_{i}$ one obtains

(A74) $$\begin{array}{c} h_{r1r1}^{\left(0\right)}\left(q\right)=4\pi C_{r1r1}^{\left(0\right)}\left(q\right)+\frac{1}{2}\rho_{0}f_{r1}C_{r1r1}^{\left(0\right)}\left(q\right)h_{r1r1}^{\left(0\right)}\left(q\right)+\frac{1}{2}\rho_{0}f_{r1}C_{r1r1}^{\left(2\right)}\left(q\right)h_{r1r1}^{\left(2\right)}\left(q\right)\\ \;+\frac{1}{2}\rho_{0}f_{r2}C_{r1r2}^{\left(0\right)}\left(q\right)h_{r1r2}^{\left(0\right)}\left(q\right)+\rho_{0}f_{r2}C_{r1r2}^{\left(2\right)}\left(q\right)h_{r1r2}^{\left(2\right)}\left(q\right)+\frac{1}{2}\rho_{0}f_{r2}C_{r1r2}^{\left(3\right)}\left(q\right)h_{r1r2}^{\left(3\right)}\left(q\right)\\ +\frac{1}{2}\rho_{0}f_{c}C_{r1c}^{\left(0\right)}\left(q\right)h_{r1c}^{\left(0\right)}\left(q\right)+\frac{1}{2}\rho_{0}f_{c}C_{r1c}^{\left(2\right)}\left(q\right)h_{r1c}^{\left(2\right)}\left(q\right), \end{array}$$

(A75) $$\begin{array}{c} h_{r2r2}^{\left(0\right)}\left(q\right)=4\pi C_{r2r2}^{\left(0\right)}\left(q\right)+\frac{1}{2}\rho_{0}f_{r2}C_{r2r2}^{\left(0\right)}\left(q\right)h_{r2r2}^{\left(0\right)}\left(q\right)+\frac{1}{2}\rho_{0}f_{r2}C_{r2r2}^{\left(2\right)}\left(q\right)h_{r2r2}^{\left(2\right)}\left(q\right)\\ \;+\frac{1}{2}\rho_{0}f_{r1}C_{r1r2}^{\left(0\right)}\left(q\right)h_{r1r2}^{\left(0\right)}\left(q\right)+\rho_{0}f_{r1}C_{r1r2}^{\left(2\right)}\left(q\right)h_{r1r2}^{\left(2\right)}\left(q\right)+\frac{1}{2}\rho_{0}f_{r1}C_{r1r2}^{\left(3\right)}\left(q\right)h_{r1r2}^{\left(3\right)}\left(q\right)\\ +\frac{1}{2}\rho_{0}f_{c}C_{r2c}^{\left(0\right)}\left(q\right)h_{r2c}^{\left(0\right)}\left(q\right)+\frac{1}{2}\rho_{0}f_{c}C_{r2c}^{\left(2\right)}\left(q\right)h_{r2c}^{\left(2\right)}\left(q\right). \end{array}$$

Multiplying Equations (A54) and (A55) by the corresponding Legendre polynomial $P_{2}\left(a_{i}\cdot k\right)$ and integrating over $a_{i}$ also yields:

(A76) $$\begin{array}{c} h_{r1r1}^{\left(2\right)}\left(q\right)=\frac{4\pi }{5}C_{r1r1}^{\left(2\right)}\left(q\right)+\frac{1}{2}\rho_{0}f_{r1}C_{r1r1}^{\left(0\right)}\left(q\right)h_{r1r1}^{\left(2\right)}\left(q\right)+\frac{1}{2}\rho_{0}f_{r1}C_{r1r1}^{\left(2\right)}\left(q\right)h_{r1r1}^{\left(5\right)}\left(q\right)\\ \;+\frac{1}{2}\rho_{0}f_{r2}C_{r1r2}^{\left(0\right)}\left(q\right)h_{r1r2}^{\left(2\right)}\left(q\right)+\frac{1}{2}\rho_{0}f_{r2}C_{r1r2}^{\left(2\right)}\left(q\right)h_{r1r2}^{\left(5\right)}\left(q\right)+\frac{1}{2}\rho_{0}f_{r2}C_{r1r2}^{\left(2\right)}\left(q\right)h_{r1r2}^{\left(7\right)}\left(q\right)\\ \;+\frac{1}{2}\rho_{0}f_{r2}C_{r1r2}^{\left(3\right)}\left(q\right)h_{r1r2}^{\left(6\right)}\left(q\right)+\frac{1}{2}\rho_{0}f_{c}C_{r1c}^{\left(0\right)}\left(q\right)h_{r1c}^{\left(2\right)}\left(q\right)+\frac{1}{2}\rho_{0}f_{c}C_{r1c}^{\left(2\right)}\left(q\right)h_{r1c}^{\left(5\right)}\left(q\right), \end{array}$$

(A77) $$\begin{array}{c} h_{r2r2}^{\left(2\right)}\left(q\right)=\frac{4\pi }{5}C_{r2r2}^{\left(2\right)}\left(q\right)+\frac{1}{2}\rho_{0}f_{r2}C_{r2r2}^{\left(0\right)}\left(q\right)h_{r2r2}^{\left(2\right)}\left(q\right)+\frac{1}{2}\rho_{0}f_{r2}C_{r2r2}^{\left(2\right)}\left(q\right)h_{r2r2}^{\left(5\right)}\left(q\right)\\ \;+\frac{1}{2}\rho_{0}f_{r1}C_{r1r2}^{\left(0\right)}\left(q\right)h_{r1r2}^{\left(2\right)}\left(q\right)+\frac{1}{2}\rho_{0}f_{r1}C_{r1r2}^{\left(2\right)}\left(q\right)h_{r1r2}^{\left(5\right)}\left(q\right)+\frac{1}{2}\rho_{0}f_{r1}C_{r1r2}^{\left(2\right)}\left(q\right)h_{r1r2}^{\left(7\right)}\left(q\right)\\ \;+\frac{1}{2}\rho_{0}f_{r1}C_{r1r2}^{\left(3\right)}\left(q\right)h_{r1r2}^{\left(6\right)}\left(q\right)+\frac{1}{2}\rho_{0}f_{c}C_{r2c}^{\left(0\right)}\left(q\right)h_{r2c}^{\left(2\right)}\left(q\right)+\frac{1}{2}\rho_{0}f_{c}C_{r2c}^{\left(2\right)}\left(q\right)h_{r2c}^{\left(5\right)}\left(q\right), \end{array}$$

where the coefficients are introduced as:

(A78) $$h_{ric}^{\left(5\right)}\left(q\right)=\frac{1}{4\pi }\int h_{ric}\left(q,a\right)P_{2}^{2}\left(a\cdot k\right)da,$$

(A79) $$h_{r1r2}^{\left(3\right)}\left(q\right)=\frac{1}{16\pi^{2}}\int h_{r1r2}\left(q,a_{1},a_{2}\right)P_{2}\left(a_{1}\cdot a_{2}\right)da_{1}da_{2}.$$

(A80) $$h_{r1r2}^{\left(7\right)}\left(q\right)=\frac{1}{16\pi^{2}}\int h_{r1r2}\left(q,a_{1},a_{2}\right)P_{2}^{2}\left(a_{1}\cdot k\right)da_{1}da_{2}.$$

As a result, we have derived a system of 12 Equations (A57) and (A60)–(A77) which are to be solved to obtain 12 expansion coefficients of the direct correlation functions: $C_{cc}^{\left(0\right)}\left(q\right)$, $C_{r1c}^{\left(0\right)}\left(q\right)$, $C_{r1c}^{\left(2\right)}\left(q\right)$, $C_{r2c}^{\left(0\right)}\left(q\right)$, $C_{r2c}^{\left(2\right)}\left(q\right)$, $C_{r1r2}^{\left(0\right)}\left(q\right)$, $C_{r1r2}^{\left(2\right)}\left(q\right)$, $C_{r1r2}^{\left(3\right)}\left(q\right)$, $C_{r1r1}^{\left(0\right)}\left(q\right)$, $C_{r1r1}^{\left(2\right)}\left(q\right)$, $C_{r2r2}^{\left(0\right)}\left(q\right)$, $C_{r2r2}^{\left(2\right)}\left(q\right)$.

## Appendix D. Density-Density Correlation Functions of Rod-Coil-Rod Polymer Chains

### Appendix D.1. Rod-Coil Total Correlation Function

Consider a correlation between the coil monomers and those of the rod of type i=1,2. We enumerate the former by $j=1,2,\ldots ,Nf_{c}$ and the latter by $k=1,2,\ldots ,Nf_{ri}$ and express:

(A81) $$\begin{array}{c} \;\langle \delta \rho_{c}\left(r_{1}\right)\delta \rho_{ri}\left(r_{3},a\right)\rangle \\ =\langle \left[\sum_{\alpha =1}^{N_{\mathrm{ch}}}\sum_{j=1}^{Nf_{c}}\delta \left(r_{1}-r_{j}^{\left(\alpha \right)}\right)-\rho_{0}f_{c}\right]\left[\sum_{\beta =1}^{N_{\mathrm{ch}}}\sum_{k=1}^{Nf_{ri}}\delta \left(r_{3}-r_{k}^{\left(\beta \right)}\right)\delta \left(a-a_{k}^{\left(\beta \right)}\right)-\frac{1}{4\pi }\rho_{0}f_{ri}\right]\rangle \\ =\langle \sum_{\alpha =1}^{N_{\mathrm{ch}}}\sum_{\beta =1}^{N_{\mathrm{ch}}}\sum_{j=1}^{Nf_{c}}\sum_{k=1}^{Nf_{ri}}\delta \left(r_{1}-r_{j}^{\left(\alpha \right)}\right)\delta \left(r_{3}-r_{k}^{\left(\beta \right)}\right)\delta \left(a-a_{k}^{\left(\beta \right)}\right)\rangle -\frac{1}{4\pi }\rho_{0}^{2}f_{c}f_{ri}\\ =N_{\mathrm{ch}}\left(N_{\mathrm{ch}}-1\right)\langle \sum_{j=1}^{Nf_{c}}\sum_{k=1}^{Nf_{ri}}\delta \left(r_{1}-r_{j}^{\left(\alpha \right)}\right)\delta \left(r_{3}-r_{k}^{\left(\beta \neq \alpha \right)}\right)\delta \left(a-a_{k}^{\left(\beta \neq \alpha \right)}\right)\rangle \\ +N_{\mathrm{ch}}\langle \sum_{j=1}^{Nf_{c}}\sum_{k=1}^{Nf_{ri}}\delta \left(r_{1}-r_{j}^{\left(\alpha \right)}\right)\delta \left(r_{3}-r_{k}^{\left(\alpha \right)}\right)\delta \left(a-a_{k}^{\left(\alpha \right)}\right)\rangle -\frac{1}{4\pi }\rho_{0}^{2}f_{c}f_{ri}\\ =N_{\mathrm{ch}}\left(N_{\mathrm{ch}}-1\right)\frac{Nf_{ri}}{4\pi V}\frac{Nf_{c}}{V}\\ +N_{\mathrm{ch}}\langle \sum_{j=1}^{Nf_{c}}\sum_{k=1}^{Nf_{ri}}\delta \left(r_{1}-r_{j}^{\left(\alpha \right)}\right)\delta \left(r_{3}-r_{k}^{\left(\alpha \right)}\right)\delta \left(a-a_{k}^{\left(\alpha \right)}\right)\rangle -\frac{1}{4\pi }\rho_{0}^{2}f_{c}f_{ri}\\ =N_{\mathrm{ch}}\left[\langle \sum_{j=1}^{Nf_{c}}\sum_{k=1}^{Nf_{ri}}\delta \left(r_{1}-r_{j}^{\left(\alpha \right)}\right)\delta \left(r_{3}-r_{k}^{\left(\alpha \right)}\right)\delta \left(a-a_{k}^{\left(\alpha \right)}\right)\rangle -\frac{N^{2}f_{c}f_{ri}}{4\pi V^{2}}\right] \end{array}$$

Accordingly, the correlator per chain reads as

(A82) $$\begin{array}{c} \;\frac{1}{N_{\mathrm{ch}}}\langle \delta \rho_{c}\left(r_{1}\right)\delta \rho_{ri}\left(r_{3},a\right)\rangle \\ =-\frac{N^{2}f_{c}f_{ri}}{4\pi V^{2}}+\sum_{i=1}^{Nf_{c}}\sum_{j=1}^{Nf_{ri}}\langle \delta \left(r_{1}-r_{j}^{\left(\alpha \right)}\right)\delta \left(r_{3}-r_{k}^{\left(\alpha \right)}\right)\delta \left(a-a_{k}^{\left(\alpha \right)}\right)\rangle , \end{array}$$

where the latter term contains averages that are the probabilities ${\tilde{P}}_{jk}\left(r_{1},r_{3},a\right)$ of finding the coil monomer *j* at the point $r_{1}$ if the rod monomer *k* is already at the point $r_{3}$ oriented along a:

(A83) $$\langle \delta \left(r_{1}-r_{j}^{\left(\alpha \right)}\right)\delta \left(r_{3}-r_{k}^{\left(\alpha \right)}\right)\delta \left(a-a_{k}^{\left(\alpha \right)}\right)\rangle ={\tilde{P}}_{jk}\left(r_{1},r_{3},a\right),$$

which is normalized as

(A84) $$\int {\tilde{P}}_{jk}\left(r_{1},r_{3}.a\right)dr_{1}dr_{3}da=1.$$

In the homogeneous phase, this probability depends only on the distance $r_{1}-r_{3}$ and we can introduce a more conventional probability

(A85) $$P_{jk}\left(r_{1}-r_{3},a\right)=V{\tilde{P}}_{jk}\left(r_{1},r_{3},a\right).$$

This enables one to obtain

(A86) $$\langle \delta \rho_{c}\left(r_{1}\right)\delta \rho_{ri}\left(r_{3},a\right)\rangle =-\frac{N\rho_{0}f_{c}f_{ri}}{4\pi V}+\frac{\rho_{0}}{N}\sum_{j=1}^{Nf_{c}}\sum_{k=1}^{Nf_{ri}}P_{jk}\left(r_{1}-r_{3},a\right).$$

At the same time, the total correlation function definition (A39) here reads

(A87) $$\langle \delta \rho_{c}\left(r_{1}\right)\delta \rho_{ri}\left(r_{3},a\right)\rangle =\frac{1}{4\pi }\rho_{0}^{2}f_{c}f_{ri}h_{ric}\left(r_{1}-r_{3},a\right),$$

and hence one obtains

(A88) $$h_{ric}\left(r,a\right)=-\frac{1}{N_{\mathrm{ch}}}+\frac{4\pi }{\rho_{0}f_{c}f_{ri}}\frac{1}{N}\sum_{j=1}^{Nf_{c}}\sum_{k=1}^{Nf_{ri}}P_{jk}\left(r,a\right),$$

where the first constant term vanishes in the thermodynamic limit.

The chain configuration probabilities can be evaluated as convolution of simpler probabilities of separate chain fragments. Indeed, let a coil monomer *j* be at the coordinate origin. Then the probability to find at a point R the end of the coil adjoined to the rod is given by the Gaussian distribution

(A89) $$G_{j0}\left(R\right)={\left(\frac{2\pi ja^{2}}{3}\right)}^{-3/2}\exp\left[-\frac{3R^{2}}{2ja^{2}}\right].$$

On the other hand, if the rod end is at the point R, its *k*-th monomer can be found at a point r only if the rod is in the right direction and r=R+kaa, i.e., with the probability

(A90) $$Q_{0k}\left(r-R,a\right)=\frac{1}{4\pi }\delta \left(r-R-kaa\right),$$

which is normalized as $\int Q_{ik}\left(r,a\right)\;dr\;da=1$.

Now the sought probability can be found by integrating over all possible coordinates of the end R:

(A91) $$\begin{array}{c} \;P_{jk}\left(r,a\right)=\int G_{j0}\left(R\right)Q_{0k}\left(r-R,a\right)dR=G_{j0}(|r-kaa|)\\ =\frac{1}{4\pi }{\left(\frac{2\pi ja^{2}}{3}\right)}^{-3/2}\exp\left(-\frac{3{\left(r-kaa\right)}^{2}}{2ja^{2}}\right), \end{array}$$

and the sum of probabilities can be approximated by the integral:

(A92) $$\frac{1}{N}\sum_{j=1}^{Nf_{c}}\sum_{k=1}^{Nf_{ri}}P_{jk}\left(r,a\right)\approx \frac{1}{4\pi N}\int_{0}^{Nf_{c}}\int_{0}^{Nf_{ri}}{\left(\frac{2\pi ja^{2}}{3}\right)}^{-3/2}\exp\left[-\frac{3{\left(r-kaa\right)}^{2}}{2ja^{2}}\right]dkdj.$$

The integral itself is difficult to take but its Fourier transform can readily be evaluated. Indeed,

(A93) $$P_{jk}\left(q,a\right)=G_{j0}\left(q\right)Q_{0k}\left(q,a\right)=\frac{1}{4\pi }\exp\left[-jq^{2}a^{2}/6\right]\cos\left[ka\left(q\cdot a\right)\right],$$

and then

(A94) $$\begin{array}{c} \;\frac{1}{N}\sum_{j=1}^{Nf_{c}}\sum_{k=1}^{Nf_{ri}}P_{jk}\left(q,a\right)\approx \frac{1}{N}\int_{0}^{Nf_{c}}\int_{0}^{Nf_{ri}}\exp\left[-jq^{2}a^{2}/6\right]\cos\left[ka\left(q\cdot a\right)\right]dkdj\\ =\frac{1}{4\pi N}\int_{0}^{Nf_{c}}\exp\left[-jq^{2}a^{2}/6\right]dj\int_{0}^{Nf_{ri}}\cos\left[ka\left(q\cdot a\right)\right]dk\\ \;=\frac{1}{4\pi x}\left[1-\exp(-f_{c}x)\right]Nf_{ri}\frac{\sin y_{i}}{y_{i}} \end{array}$$

where $y_{i}=Nf_{ri}a\left(q\cdot a\right)$. Substituting this into Equation (A88), one obtains $h_{ric}\left(q,a\right)$ in the form of Equation (32).

### Appendix D.2. Total Correlation Function of Segments of the Same Rod

Let us consider any two segments which belong to the same rod of the same chain. These segments are not truly statistically independent being always aligned in the same direction. The monomers in different chains are not restricted and we can formally consider

(A95) $$\begin{array}{c} \;\langle \delta \rho_{ri}\left(r_{1},a_{1}\right)\delta \rho_{ri}\left(r_{3},a_{3}\right)\rangle \\ =\langle \left[\sum_{\alpha =1}^{N_{\mathrm{ch}}}\sum_{j=1}^{Nf_{ri}}\delta \left(r_{1}-r_{j}^{\left(\alpha \right)}\right)\delta \left(a_{1}-a^{\left(\alpha \right)}\right)-\frac{1}{4\pi }\rho_{0}f_{ri}\right]\\ \times \left[\sum_{\beta =1}^{N_{\mathrm{ch}}}\sum_{k=1}^{Nf_{ri}}\delta \left(r_{3}-r_{k}^{\left(\beta \right)}\right)\delta \left(a_{3}-a^{\left(\beta \right)}\right)-\frac{1}{4\pi }\rho_{0}f_{ri}\right]\rangle \\ =\langle \sum_{\alpha =1}^{N_{\mathrm{ch}}}\sum_{\beta =1}^{N_{\mathrm{ch}}}\sum_{j=1}^{Nf_{ri}}\sum_{k=1}^{Nf_{ri}}\delta \left(r_{1}-r_{j}^{\left(\alpha \right)}\right)\delta \left(r_{3}-r_{k}^{\left(\beta \right)}\right)\delta \left(a_{1}-a^{\left(\alpha \right)}\right)\delta \left(a_{3}-a^{\left(\beta \right)}\right)\rangle -\frac{1}{16\pi^{2}}\rho_{0}^{2}f_{ri}^{2}\\ =N_{\mathrm{ch}}\left(N_{\mathrm{ch}}-1\right)\langle \sum_{j=1}^{Nf_{ri}}\sum_{k=1}^{Nf_{ri}}\delta \left(r_{1}-r_{j}^{\left(\alpha \right)}\right)\delta \left(a_{1}-a^{\left(\alpha \right)}\right)\delta \left(r_{3}-r_{k}^{\left(\beta \neq \alpha \right)}\right)\delta \left(a_{3}-a_{k}^{\left(\beta \neq \alpha \right)}\right)\rangle \\ +N_{\mathrm{ch}}\langle \sum_{j=1}^{Nf_{ri}}\sum_{k=1}^{Nf_{ri}}\delta \left(r_{1}-r_{j}^{\left(\alpha \right)}\right)\delta \left(r_{3}-r_{k}^{\left(\alpha \right)}\right)\delta \left(a_{1}-a^{\left(\alpha \right)}\right)\delta \left(a_{3}-a^{\left(\alpha \right)}\right)\rangle -\frac{1}{16\pi^{2}}\rho_{0}^{2}f_{ri}^{2}\\ =N_{\mathrm{ch}}\left(N_{\mathrm{ch}}-1\right)\frac{N^{2}f_{ri}^{2}}{16\pi^{2}V^{2}}\\ +N_{\mathrm{ch}}\langle \sum_{j=1}^{Nf_{ri}}\sum_{k=1}^{Nf_{ri}}\delta \left(r_{1}-r_{j}^{\left(\alpha \right)}\right)\delta \left(r_{3}-r_{k}^{\left(\alpha \right)}\right)\delta \left(a_{1}-a^{\left(\alpha \right)}\right)\delta \left(a_{3}-a^{\left(\alpha \right)}\right)\rangle -\frac{1}{16\pi^{2}}\rho_{0}^{2}f_{ri}^{2}\\ =N_{\mathrm{ch}}\left[\langle \sum_{j=1}^{Nf_{ri}}\sum_{k=1}^{Nf_{ri}}\delta \left(r_{1}-r_{j}^{\left(\alpha \right)}\right)\delta \left(r_{3}-r_{k}^{\left(\alpha \right)}\right)\delta \left(a_{1}-a^{\left(\alpha \right)}\right)\delta \left(a_{3}-a^{\left(\alpha \right)}\right)\rangle -\frac{N^{2}f_{ri}^{2}}{16\pi^{2}V^{2}}\right] \end{array}$$

Similar to the previous subsection, we write the expression for the correlator per chain as a sum of two terms. The first term is a sum over all segments while the second term describes correlations between different segments:

(A96) $$\begin{array}{c} \;\frac{1}{N_{\mathrm{ch}}}\langle \delta \rho_{ri}\left(r_{1},a_{1}\right)\delta \rho_{ri}\left(r_{3},a_{3}\right)\rangle \\ =\sum_{j=1}^{Nf_{ri}}\sum_{k=1}^{Nf_{ri}}\langle \delta \left(r_{1}-r_{j}^{\left(\alpha \right)}\right)\delta \left(r_{3}-r_{k}^{\left(\alpha \right)}\right)\delta \left(a_{1}-a^{\left(\alpha \right)}\right)\delta \left(a_{3}-a^{\left(\alpha \right)}\right)\rangle -\frac{N^{2}f_{ri}^{2}}{16\pi^{2}V^{2}}\\ =\sum_{j=1}^{Nf_{ri}}\langle \delta \left(r_{1}-r_{j}^{\left(\alpha \right)}\right)\delta \left(r_{3}-r_{j}^{\left(\alpha \right)}\right)\delta \left(a_{1}-a^{\left(\alpha \right)}\right)\delta \left(a_{3}-a^{\left(\alpha \right)}\right)\rangle \\ +\sum_{j\neq k}^{Nf_{ri}}\langle \delta \left(r_{1}-r_{j}^{\left(\alpha \right)}\right)\delta \left(r_{3}-r_{k}^{\left(\alpha \right)}\right)\delta \left(a_{1}-a^{\left(\alpha \right)}\right)\delta \left(a_{3}-a^{\left(\alpha \right)}\right)\rangle -\frac{N^{2}f_{ri}^{2}}{16\pi^{2}V^{2}}\\ =\frac{Nf_{ri}}{4\pi V}\delta \left(r_{3}-r_{1}\right)\delta \left(a_{3}-a_{1}\right)+\sum_{j\neq k}^{Nf_{ri}}{\tilde{P}}_{jk}\left(r_{1},r_{3},a_{1},a_{3}\right)-\frac{N^{2}f_{ri}^{2}}{16\pi^{2}V^{2}}, \end{array}$$

where ${\tilde{P}}_{jk}\left(r_{1},r_{3},a_{1},a_{3}\right)$ is the probability to find *j*-th and *k*-th monomers of the same rod at corresponding coordinates and with the corresponding orientations. It is formally normalized as

(A97) $$\int {\tilde{P}}_{jk}\left(r_{1},r_{3},a_{1},a_{3}\right)dr_{1}dr_{3}da_{1}da_{3}=1,$$

and can be expressed in a simplified form accounting for the rod linear structure:

(A98) $${\tilde{P}}_{jk}\left(r_{1},r_{3},a_{1},a_{3}\right)=\delta \left(a_{3}-a_{1}\right)\frac{1}{V}Q_{jk}\left(r_{1}-r_{3},a_{1}\right),$$

where $Q_{jk}\left(r,a\right)$ is (as previously) the probability to find *j*-th monomer at point r aligned along a if *k*-th monomer is at the origin being similarly aligned. This enables one to express the correlator as

(A99) $$\begin{array}{c} \langle \delta \rho_{ri}\left(r_{1},a_{1}\right)\delta \rho_{ri}\left(r_{3},a_{3}\right)\rangle \\ \;=\frac{\rho_{0}f_{ri}}{4\pi }\delta \left(r_{3}-r_{1}\right)\delta \left(a_{3}-a_{1}\right)+\delta \left(a_{3}-a_{1}\right)\frac{\rho_{0}}{N}\sum_{j\neq k}^{Nf_{ri}}Q_{jk}\left(r_{1}-r_{3},a_{1}\right)-\frac{\rho_{0}^{2}f_{ri}^{2}}{16\pi^{2}N_{\mathrm{ch}}}, \end{array}$$

On the other hand, the total correlation function definition (A39) here can be written in the form:

(A100) $$\langle \delta \rho_{ri}\left(r_{1},a_{1}\right)\delta \rho_{ri}\left(r_{3},a_{3}\right)\rangle =\frac{\rho_{0}f_{ri}}{4\pi }\delta \left(r_{1}-r_{3}\right)\delta \left(a_{1}-a_{3}\right)+\frac{\rho_{0}^{2}f_{ri}^{2}}{16\pi^{2}}h_{riri}\left(r_{1},r_{3},a_{1},a_{3}\right),$$

and (again in the limit $N_{\mathrm{ch}}\to \infty$) one obtains:

(A101) $$h_{riri}\left(r_{1},r_{3},a_{1},a_{3}\right)=\delta \left(a_{3}-a_{1}\right)h_{riri}\left(r_{1}-r_{3},a_{1}\right),$$

where

(A102) $$h_{riri}\left(r,a\right)=\frac{16\pi^{2}}{\rho_{0}f_{ri}^{2}}\frac{1}{N}\sum_{j\neq k}^{Nf_{ri}}Q_{jk}\left(r,a\right).$$

Taking into account that

(A103) $$Q_{jk}\left(r,a\right)=\frac{1}{4\pi }\delta \left(r-\left(k-j\right)aa\right),$$

we finally express the cosine Fourier harmonic of the correlation function as given by Equation (33).

### Appendix D.3. Total Correlation Function between Segments of the Two Different Rods

For the monomers belonging to the rods of different type we introduce the orientation of the rods of type 1 and type 2 of a chain α as $a^{\left(1\alpha \right)}$ and $a^{\left(2\alpha \right)}$ and write the correlator as:

(A104) $$\begin{array}{c} \;\langle \delta \rho_{r1}\left(r_{1},a_{1}\right)\delta \rho_{r2}\left(r_{3},a_{3}\right)\rangle \\ =\langle \left[\sum_{\alpha =1}^{N_{\mathrm{ch}}}\sum_{j=1}^{Nf_{r1}}\delta \left(r_{1}-r_{j}^{\left(\alpha \right)}\right)\delta \left(a_{1}-a^{\left(1\alpha \right)}\right)-\frac{1}{4\pi }\rho_{0}f_{r1}\right]\\ \times \left[\sum_{\beta =1}^{N_{\mathrm{ch}}}\sum_{k=1}^{Nf_{r2}}\delta \left(r_{3}-r_{k}^{\left(\beta \right)}\right)\delta \left(a_{3}-a^{\left(2\beta \right)}\right)-\frac{1}{4\pi }\rho_{0}f_{r2}\right]\rangle \\ =\langle \sum_{\alpha =1}^{N_{\mathrm{ch}}}\sum_{\beta =1}^{N_{\mathrm{ch}}}\sum_{j=1}^{Nf_{r1}}\sum_{k=1}^{Nf_{r2}}\delta \left(r_{1}-r_{j}^{\left(\alpha \right)}\right)\delta \left(r_{3}-r_{k}^{\left(\beta \right)}\right)\delta \left(a_{1}-a^{\left(1\alpha \right)}\right)\delta \left(a_{3}-a^{\left(2\beta \right)}\right)\rangle -\frac{1}{16\pi^{2}}\rho_{0}^{2}f_{r1}f_{r2}\\ =N_{\mathrm{ch}}\left(N_{\mathrm{ch}}-1\right)\langle \sum_{j=1}^{Nf_{r1}}\sum_{k=1}^{Nf_{r2}}\delta \left(r_{1}-r_{j}^{\left(\alpha \right)}\right)\delta \left(a_{1}-a^{\left(1\alpha \right)}\right)\delta \left(r_{3}-r_{k}^{\left(\beta \neq \alpha \right)}\right)\delta \left(a_{3}-a_{k}^{\left(2\beta \neq 2\alpha \right)}\right)\rangle \\ +N_{\mathrm{ch}}\langle \sum_{j=1}^{Nf_{r1}}\sum_{k=1}^{Nf_{r2}}\delta \left(r_{1}-r_{j}^{\left(\alpha \right)}\right)\delta \left(r_{3}-r_{k}^{\left(\alpha \right)}\right)\delta \left(a_{1}-a^{\left(1\alpha \right)}\right)\delta \left(a_{3}-a^{\left(2\alpha \right)}\right)\rangle -\frac{1}{16\pi^{2}}\rho_{0}^{2}f_{r1}f_{r2}\\ =N_{\mathrm{ch}}\left(N_{\mathrm{ch}}-1\right)\frac{N^{2}f_{r1}f_{r2}}{16\pi^{2}V^{2}}\\ +N_{\mathrm{ch}}\langle \sum_{j=1}^{Nf_{r1}}\sum_{k=1}^{Nf_{r2}}\delta \left(r_{1}-r_{j}^{\left(\alpha \right)}\right)\delta \left(r_{3}-r_{k}^{\left(\alpha \right)}\right)\delta \left(a_{1}-a^{\left(1\alpha \right)}\right)\delta \left(a_{3}-a^{\left(2\alpha \right)}\right)\rangle -\frac{1}{16\pi^{2}}\rho_{0}^{2}f_{r1}f_{r2}\\ =N_{\mathrm{ch}}\left[\langle \sum_{j=1}^{Nf_{r1}}\sum_{k=1}^{Nf_{r2}}\delta \left(r_{1}-r_{j}^{\left(\alpha \right)}\right)\delta \left(r_{3}-r_{k}^{\left(\alpha \right)}\right)\delta \left(a_{1}-a^{\left(1\alpha \right)}\right)\delta \left(a_{3}-a^{\left(2\alpha \right)}\right)\rangle -\frac{N^{2}f_{r1}f_{r2}}{16\pi^{2}V^{2}}\right] \end{array}$$

Taking into account that in the sums above all terms correspond to different segments one obtains:

(A105) $$\begin{array}{c} \;\frac{1}{N_{\mathrm{ch}}}\langle \delta \rho_{r1}\left(r_{1},a_{1}\right)\delta \rho_{r2}\left(r_{3},a_{3}\right)\rangle \\ \;=\sum_{j=1}^{Nf_{r1}}\sum_{k=1}^{Nf_{r2}}\langle \delta \left(r_{1}-r_{j}^{\left(\alpha \right)}\right)\delta \left(r_{3}-r_{k}^{\left(\alpha \right)}\right)\delta \left(a_{1}-a^{\left(1\alpha \right)}\right)\delta \left(a_{3}-a^{\left(2\alpha \right)}\right)\rangle -\frac{N^{2}f_{r1}f_{r2}}{16\pi^{2}V^{2}}\\ \;=\sum_{j=1}^{Nf_{r1}}\sum_{k=1}^{Nf_{r2}}{\tilde{P}}_{jk}\left(r_{1},r_{3},a_{1},a_{3}\right)-\frac{N^{2}f_{r1}f_{r2}}{16\pi^{2}V^{2}}, \end{array}$$

where ${\tilde{P}}_{jk}\left(r_{1},r_{3},a_{1},a_{3}\right)$ is the probability to find *j*-th monomer of type 1 and *k*-th monomer of type 2 at the corresponding coordinates and with the corresponding orientations. It is formally normalized as

(A106) $$\int {\tilde{P}}_{jk}\left(r_{1},r_{3},a_{1},a_{3}\right)dr_{1}dr_{3}da_{1}da_{3}=1,$$

and in the disordered phase can be expressed as:

(A107) $${\tilde{P}}_{jk}\left(r_{1},r_{3},a_{1},a_{3}\right)=\frac{1}{V}P_{jk}\left(r_{1}-r_{3},a_{1},a_{3}\right),$$

where $P_{jk}\left(r,a\right)$ is the probability to find *j*-th monomer of type 1 at point r aligned along $a_{1}$ if *k*-th monomer of type 2 is at the origin being aligned along $a_{3}$. This allows expressing the correlator as

(A108) $$\langle \delta \rho_{ri}\left(r_{1},a_{1}\right)\delta \rho_{ri}\left(r_{3},a_{3}\right)\rangle =\frac{\rho_{0}}{N}\sum_{j=1}^{Nf_{r1}}\sum_{k=1}^{Nf_{r2}}P_{jk}\left(r_{1}-r_{3},a_{1},a_{3}\right)-\frac{\rho_{0}^{2}f_{r1}f_{r2}}{16\pi^{2}N_{\mathrm{ch}}},$$

The corresponding total correlation function definition (A39) yields:

(A109) $$\langle \delta \rho_{r1}\left(r_{1},a_{1}\right)\delta \rho_{r2}\left(r_{3},a_{3}\right)\rangle =\frac{\rho_{0}^{2}f_{r1}f_{r2}}{16\pi^{2}}h_{r1r2}\left(r_{1}-r_{3},a_{1},a_{3}\right),$$

and in the thermodynamic limit $N_{\mathrm{ch}}\to \infty$ one obtains the following expression:

(A110) $$h_{r1r2}\left(r,a_{1},a_{3}\right)=\frac{16\pi^{2}}{\rho_{0}f_{r1}f_{r2}}\frac{1}{N}\sum_{j=1}^{Nf_{r1}}\sum_{k=1}^{Nf_{r2}}P_{jk}\left(r,a_{1},a_{3}\right).$$

The probability here can again be written as a convolution of simpler probabilities if we suppose that the monomer *k* is at the origin and denote as $R_{1}$ and $R_{2}$ the coordinates of the coil ending monomers:

(A111) $$P_{jk}\left(r,a_{1},a_{3}\right)=\int Q_{j0}\left(r-R_{1},a_{1}\right)G_{0Nf_{c}}\left(\right|R_{1}-R_{2}\left|\right)Q_{0k}\left(R_{2},a_{3}\right)dR_{1}dR_{2},$$

This integral can again be readily evaluated for the corresponding Fourier transform.

Indeed, the integral convolution automatically reduces to the product of exponential Fourier transforms:

(A112) $$P_{jk}^{\exp}\left(q,a_{1},a_{3}\right)=\int P_{jk}\left(r,a_{1},a_{3}\right)\exp\left(-iq\cdot r\right)dr=Q_{j0}^{\exp}\left(q,a_{1}\right)G_{0Nf_{c}}^{\exp}\left(q\right)Q_{0k}^{exp}\left(q,a_{3}\right),$$

where

(A113) $$Q_{jk}^{\exp}\left(q,a\right)=\int Q_{jk}\left(r,a\right)\exp\left(-iq\cdot r\right)dr=\frac{1}{4\pi }\exp\left[-i\left(k-j\right)a\left(q\cdot a\right)\right].$$

The coil probability distribution (A89) is an even function of the coordinates and its exponential Fourier transform reads:

(A114) $$P_{0Nf_{r}}^{\exp}\left(q\right)=\int P_{0Nf_{r}}\left(r\right)\exp\left(-iq\cdot r\right)dr=\exp\left(-f_{c}x\right).$$

Accordingly, the cosine Fourier transform of the rod-rod probability can be expressed as:

(A115) Pjk(q,a1,a3)=RePjkexp(q,a1,a3)=116π2exp(−fcx)cos[a(ja1−ka3)·q].

As a result, we obtain the cosine Fourier transform of the total correlation function between segments of the two different rods separated by a coil, $h_{r1r2}\left(q,a_{1},a_{2}\right)$, as given by Equation (34).
